# Cascade‐targeted intervention of the NLRP3 inflammasome and downstream angiogenesis by curcumin nanoparticles for ocular neovascularization therapy

**DOI:** 10.1002/ctm2.70846

**Published:** 2026-09-21

**Authors:** Siyu Jiang, Ziwei Cheng, Yujuan Cai, Yanji Zhu, Nyamjargal Nyambayar, Ruijie Lin, Li Zhang, Jian Chen, Bing Xie, Xiuping Chen

**Affiliations:** ^1^ Department of Ophthalmology Ruijin Hospital Shanghai Jiao Tong University School of Medicine Shanghai China; ^2^ Engineering Research Center of Cell and Therapeutic Antibody Ministry of Education School of Pharmacy Shanghai Jiao Tong University Shanghai China; ^3^ Instrumental Analysis Center Shanghai Jiao Tong University Shanghai China; ^4^ Crean Lutheran High School Irvine California USA; ^5^ Department of Ophthalmology Zhongshan Hospital Fudan University Shanghai China

**Keywords:** anti‐angiogenesis therapy, curcumin nanoparticles, NLRP3 inflammasome, retinal neovascularization

## Abstract

**Background:**

Retinal neovascularization (RNV) causes irreversible vision loss. Despite the benefits of anti‐VEGF therapy, incomplete responses and persistent inflammation necessitate complementary treatments. Curcumin has anti‐inflammatory and anti‐angiogenic activity, but its poor solubility and bioavailability limit ocular application.

**Methods:**

PLGA‐encapsulated curcumin nanoparticles (Cur‐NPs; approximately 125 nm) were produced using an ultrasound‐microreactor platform. Therapeutic efficacy was assessed in oxygen‐induced retinopathy (OIR) and Rho/VEGF transgenic mouse models following intravitreal administration. Neovascularization, vascular leakage, retinal structure, apoptosis, retinal distribution, and intraocular retention were evaluated using histology, fluorescence imaging, and longitudinal in vivo imaging. Mechanisms were investigated using single‐cell transcriptomics, untargeted metabolomics, genetic loss‐of‐function, and pharmacological perturbation. Conbercept monotherapy and Cur‐NP combination treatment were also evaluated.

**Results:**

Cur‐NPs reduced pathological neovascularization in both models and improved vascular integrity, decreased vascular leakage, preserved retinal architecture, and attenuated apoptosis in OIR mice. Compared with free curcumin, Cur‐NPs showed broader retinal fluorescence distribution and prolonged intraocular fluorescence retention. Cur‐NPs suppressed the NLRP3ASCCaspase‐1 inflammasome and associated VEGF/VEGFR2 signaling. Genetic and pharmacological experiments supported a functional contribution of NLRP3 signaling to the anti‐angiogenic effects of Cur‐NPs. Multi‐omics analyses revealed treatment‐associated changes in retinal immune‐cell states and metabolic pathways. ConberceptCur‐NP combination therapy reduced neovascularization more than either treatment alone.

**Conclusions:**

Cur‐NPs inhibit pathological RNV through modulation of NLRP3‐associated inflammatoryangiogenic signaling while improving retinal protection and intraocular retention. These findings support further evaluation of Cur‐NPs as a potential upstream anti‐inflammatory adjunct to VEGF‐directed therapy.

## Key points

- Cur‐NPs suppress pathological retinal neovascularization and vascular leakage in two complementary murine models.
- Cur‐NPs preserve retinal vascular integrity and architecture while reducing retinal apoptosis.
- Inhibition of the NLRP3ASCCaspase‐1 inflammasome is associated with attenuation of VEGF‐related angiogenic signaling.
- Single‐cell transcriptomic and metabolomic analyses reveal inflammatory and metabolic remodeling of the retinal microenvironment.

## Graphical abstract

1. Ultrasound‐microreactor synthesizes monodisperse 125 nm Cur‐NPs with enhanced solubility.
2. Cur‐NPs suppress VEGF signaling and retinal neovascularization via the NLRP3 axis.
3. Multi‐omics profiling reveals that Cur‐NPs reprogram retinal microglia and metabolic pathways.
4. PLGA‐based microreactor synthesis enables scalable clinical translation for RNV therapy.

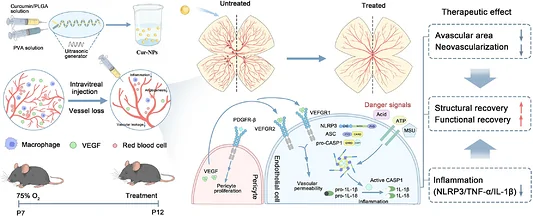

## 1 INTRODUCTION

Retinal neovascularization (RNV) is a defining pathological characteristic of several major blinding ocular disorders. This is clinically exemplified by conditions such as diabetic retinopathy (DR), neovascular age‐related macular degeneration (nAMD), and retinal vein occlusion (RVO).^1^, ^2^, ^3^, ^4^, ^5^ These conditions are underpinned by pathological hallmarks such as dysregulated angiogenesis and compromise of the blood‐retinal barrier (BRB), which culminate in severe vision‐impairing complications, including intraretinal/subretinal oedema, vitreous haemorrhage and tractional retinal detachment.^6^, ^7^, ^8^, ^9^ Despite being the mainstay of clinical management, current anti‐VEGF therapies are encumbered by several limitations, including the need for frequent intravitreal injections (typically at intervals of 4–8 weeks), the emergence of treatment resistance (documented in 10%–40% of cases), substantial healthcare burdens, and potential systemic side effects. These factors collectively underscore the urgent need for alternative therapeutic approaches that target upstream inflammatory drivers.^10^, ^11^, ^12^, ^13^, ^14^

Curcumin, a natural polyphenol derived from Curcuma longa, holds considerable promise as a pleiotropic therapeutic agent due to its broad‐spectrum anti‐inflammatory, antioxidant, and anti‐angiogenic properties.^15^, ^16^, ^17^, ^18^ Compared to bioactive molecules that target a single pathway, the distinct advantage of curcumin stems from its pleiotropic regulatory profile, which allows for the sequential modulation of the upstream inflammatory (specifically NLRP3‐mediated) cascade and the consequent angiogenic (VEGF‐mediated) signalling. This cascade‐targeted profile is particularly potent in disrupting the reciprocal feedback loop between chronic inflammation and pathological vessel growth, thereby potentially overcoming the limitations of current monotherapies. However, its clinical translation is significantly curtailed by its poor aqueous solubility (0.6 µg/mL), rapid systemic clearance (plasma half‐life < 1 h), and negligible oral bioavailability (1%).^19^, ^20^, ^21^, ^22^ To address these pharmacokinetic hurdles, we have developed poly (lactic‐co‐glycolic acid) (PLGA)‐encapsulated curcumin nanoparticles (Cur‐NPs). Notably, the extreme hydrophobicity of curcumin makes it an ideal candidate for PLGA‐based encapsulation, facilitating high drug loading within the hydrophobic core of the polymer matrix, thereby ensuring structural stability and enabling the prolonged retention characteristics necessary to reach and persist within the posterior segment. Earlier work by Pradhan et al. demonstrated that curcumin nanoparticles can effectively inhibit LPS‐induced NF‐κB signaling in corneal cells, leading to suppressed VEGF expression and subsequent mitigation of corneal neovascularization.^23^ These findings underscore the comprehensive benefits of Cur‐NPs: they retain curcumin's native pleiotropic properties while successfully circumventing its pharmacokinetic limitations.^24^, ^25^

Notably, while the effects of curcumin on corneal neovascularization have been relatively well explored,^26^, ^27^, ^28^ its potential in treating the anatomically and physiologically complex retinal and choroidal neovascularization remains underinvestigated.^29^, ^30^ This represents a critical knowledge gap, especially given the emerging recognition of the NLRP3 inflammasome as a key regulator of RNV pathogenesis.^31^, ^32^, ^33^, ^34^, ^35^ The NLRP3 inflammasome activation cascade leads to caspase‐1‐mediated maturation of IL‐1β and IL‐18, creating a pro‐angiogenic inflammatory microenvironment that exacerbates retinal vascular pathology.^36^, ^37^ Crucially, cytokines such as IL‐1β and IL‐18 can directly stimulate VEGF production within pericytes as well as human retinal microvascular endothelial cells (HRMVECs)—the major cellular constituents that form the blood–retinal barrier (BRB)—thereby driving pathological vessel sprouting. Curcumin has exhibited promising inhibitory effects on the NLRP3 inflammasome,^38^, ^39^, ^40^ suggesting a potential novel therapeutic pathway for disrupting this upstream–downstream cascade and mitigating RNV by stabilizing the retinal microenvironment.

This study aims to: (1) conduct a comprehensive evaluation of the therapeutic potential of Cur‐NPs in established in vivo and in vitro RNV models, and (2) elucidate their mechanism of action through the modulation of the NLRP3 inflammasome and its subsequent impact on downstream angiogenic pathways. The clinical significance of this study is twofold. First, it addresses the unmet clinical need for therapeutics targeting the intertwined inflammatory‐angiogenic axis of RNV. Second, it establishes a translational foundation for the development of curcumin formulations with enhanced solubility and sustained‐release properties, which could serve as adjuncts to current anti‐VEGF therapies. Our findings may pave the way for novel, cascade‐targeted therapeutic strategies that integrate anti‐inflammatory and anti‐angiogenic approaches for retinal vascular diseases.

## 2 MATERIALS AND METHODS

### 2.1 Preparation and characterization of the Cur‐NPs

PLGA (50:50, molecular weight, inherent viscosity: 0.2 dL/g) was purchased from Shenzhen LvBao Technology Co., Ltd. Curcumin (≥98% purity) was obtained from Shanghai Adamas Reagent Co., Ltd. PVA 0588 (alcoholysis degree: 87%‐89%) was acquired from Shanghai Macklin Biochemical Technology Co., Ltd. Ethyl acetate of analytical grade was supplied by Shanghai Titan Technology Co., Ltd.

An emulsion‐solvent evaporation approach was employed to synthesize Cur‐NPs within an ultrasonic microreactor (UM‐Flow, MoGe Technology Co., Ltd.). Curcumin and PLGA were dissolved in ethyl acetate to form the organic phase, while a 3% (w/v) PVA solution served as the aqueous phase. The organic and aqueous phases were infused into the ultrasonic microreactor via syringe pumps at a flow rate ratio of 1:5.4, maintaining a total flow rate of 0.54 mL/min. Ultrasonication was performed at 40 W power. The resulting emulsion was collected, and the organic solvent was subsequently evaporated using a centrifugal vacuum concentrator (40°C, 10 mbar, 40 min). The nanoparticles were then purified by ultrafiltration centrifugation, and the curcumin concentration was determined using an ultraviolet‐visible spectrophotometer (EV300, Thermo Scientific) at 426 nm.

The particle size distribution and zeta potential of the PLGA nanoparticles were analysed using a NanoBrook Omni particle size and zeta potential analyser (Brookhaven Instruments). For morphological characterization, a minuscule droplet of the nanoparticle suspension was applied onto a carbon‐coated copper grid for a 1‐minute adsorption period. Residual liquid was gently blotted away. Under light protection conditions, the sample was stained with 1% phosphotungstic acid for 1 min, and then the phosphotungstic acid was removed. After drying, the sample was imaged using a transmission electron microscope (TEM, Talos F200C G2).

An appropriate amount of Cur‐NPs was lyophilized and weighed to determine the total mass of the nanoparticles. An equivalent volume of the sample was transferred into an ultrafiltration tube to separate the Cur‐NPs from the free drug. The concentration of curcumin retained in the inner compartment, as well as the concentration in the original non‐ultrafiltered Cur‐NPs, was measured at 426 nm using an ultraviolet‐visible (UV‐Vis) spectrophotometer. The EE and DL were calculated using the following formulas:

$$\mathrm{EE}\left(\%\right)=\left(\frac{\mathrm{Weight}\;\mathrm{of}\;\mathrm{encapsulated}\;\mathrm{curcumin}}{\mathrm{Weight}\;\mathrm{of}\;\mathrm{total}\;\mathrm{curcumin}\;\mathrm{added}}\right)\times 100\%$$

$$\mathrm{DL}(\%)=\left(\frac{\mathrm{Weight}\mathrm{of}\;\mathrm{encapsulated}\;\mathrm{curcumin}}{\mathrm{Weight}\;\mathrm{of}\;\mathrm{lyophilized}\;\mathrm{nanoparticles}}\right)\times 100\%$$

To assess the in vitro release profile of curcumin, 5 mL of Cur‐NP suspension was loaded into a 10 kDa dialysis bag, which was immersed in 100 ml of release medium (PBS buffer, pH 7.4, containing 2% Tween‐80). The system was incubated at 37°C with continuous shaking at 100 rpm. At predetermined time intervals, samples were collected and replaced with an equal volume of fresh release medium to maintain sink conditions. The curcumin concentration in the samples was determined by UPLC as previously described,^41^ and the cumulative release was calculated based on the measured drug concentrations.

### 2.2 Animals and ethics statement

Specific pathogen‐free (SPF) C57BL/6 mice (Charles River Laboratories) and rhodopsin promoter/VEGF (rho/VEGF) transgenic mice^42^ were used in this study. All mice were housed in a barrier SPF facility under standardized environmental conditions. We executed all live‐animal procedures in strict adherence to the guidelines outlined in the Guide for the Care and Use of Laboratory Animals. The experimental protocol received approval from the Scientific Investigation Board of Shanghai Jiao Tong University School of Medicine (Shanghai, China).

### 2.3 Establishment of an oxygen‐induced retinal ischemia model in mice

Neonatal mice at postnatal Day 7 (P7), along with their nursing mothers, were exposed to 75% ± 2% oxygen for 5 days and then returned to room air at P12 to induce retinal ischemia and pathological neovascularization.^43^ At P12, one eye of each mouse received an intravitreal injection of 1 µL Cur‐NPs at varying concentrations (0.01, 0.05, 0.1 or 0.5 µg/µL) using a Hamilton syringe with a 33‐gauge needle, while the contralateral eye was administered 1 µL sterile PBS as a control. Ocular samples were collected at P18, a fully established stage of extensive retinal neovascularization, for histological, molecular, and biochemical analyses.

### 2.4 Retinal flat‐mount staining and quantification of retinal neovascularization

Following deep anaesthesia and euthanasia, the eyes were rapidly enucleated and fixed in 4% paraformaldehyde (PFA) at room temperature for 1 h, followed by a rinse with PBS. Under a stereomicroscope, a circumferential incision was made along the corneal limbus to remove the cornea, lens, and vitreous body. The intact retina was gently dissected from the surrounding ocular tissues and radially incised to facilitate flat‐mount preparation. The isolated retinas were incubated with GSL I isolectin B4 (IB4; 1:50 dilution in PBS) at room temperature for 45–60 min in the dark and subsequently washed three times with PBS (10 min each). For OIR mice, the stained retinas were placed on glass slides with the vitreal surface facing upward, and four to five radial incisions were made from the peripheral retina toward the optic nerve head to gently flatten the tissue with fine forceps while avoiding mechanical damage. After removal of excess liquid, antifade mounting medium was added, and the retina was covered with a coverslip, with the four corners secured using clear nail polish.^44^ For Rho/VEGF transgenic mice, because neovascular lesions are predominantly localized to the subretinal space between the neural retina and the RPE/choroid complex, the retinal tissue was cut and flattened directly on a coverslip, followed by mounting with antifade medium on a glass slide to facilitate visualization of subretinal neovascularization (sub‐RNV) under an upright fluorescence microscope. Fluorescence images were acquired in the green channel for IB4 staining. For each retina, the entire retinal vascular network was systematically imaged using a 4× objective, with adjacent fields captured with at least 30% overlap to enable subsequent panoramic reconstruction. Representative neovascular lesions were additionally imaged using a 20× objective for detailed morphological assessment. The acquired images were stitched using Adobe Photoshop to generate whole‐retina panoramic montages, which were subsequently analyzed using Image‐Pro Plus 6.0 (Media Cybernetics, USA). For OIR retinas, the total RNV area (mm^2^) was quantified from the reconstructed whole‐retina montages, whereas for Rho/VEGF transgenic retinas, the total number of subretinal neovascular buds was quantified.

### 2.5 Immunofluorescence staining

Paraffin‐embedded retinal sections were deparaffinized and rehydrated using an environmentally friendly dewaxing solution (G1128, Servicebio) and ethanol. Antigen retrieval was performed with EDTA antigen repair solution (pH 8.0, G1206, Servicebio) for 30 min at 37°C. Endogenous peroxidase was blocked using 3% hydrogen peroxide (Angergech) for 25 minutes. Sections were incubated with primary antibodies against F4/80 (GB113373, Servicebio), NLRP3 (GB114320, Servicebio), PDGFR‐β (SC374573, Santa Cruz Biotechnology), and CD31 (GB11063‐2, Servicebio) diluted in sterile PBS (G4250, Servicebio) overnight at 4°C. Following PBS washes, tissue sections were exposed to HRP‐labelled secondary antibodies (GB23303, Servicebio) and maintained at room temperature for 50 min, followed by TSA amplification using iF647‐Tyramide (G1232, Servicebio) for 10 minutes. DAPI (G1012, Servicebio) was used for nuclear counterstaining.^45^ Sections were then treated with tissue autofluorescence quencher (G1221, Servicebio). Following sealing with an anti‐fade mounting matrix (G1401, Servicebio), fluorophore visualization was subsequently executed via a specialized fluorescence microscopy system.

### 2.6 Evans blue assay

Mice received intraperitoneal injections of 0.3 mL 1% Evans blue solution. After 3 h of systemic circulation (confirmed by visible blue discoloration of peripheral tissues, including lips, ears and paws), animals were euthanized, and eyes were immediately enucleated. Retinal tissues were fixed in 4% paraformaldehyde (60 min, room temperature), flat‐mounted on glass slides under stereomicroscopic guidance, and protected with anti‐fade mounting medium. Vascular leakage was assessed using fluorescence microscopy (Eclipse Ni‐U, Nikon).^46^ Quantitative analysis of extravasated Evans blue was performed by batch‐processing retinal images with customized Image‐Pro Plus macros (v6.0, Media Cybernetics), with fluorescence intensity normalized to background levels.

### 2.7 HE staining

For histological evaluation, paraffin‐embedded tissue slices underwent sequential deparaffinization utilizing an eco‐friendly dewaxing transparent liquid (I and II; 20 min per stage). This was succeeded by a rehydration cascade through descending ethanol grades (100% I/II for 5 min each, followed by 75% for 5 min) before a final rinse in tap water. Sections were pretreated with HD buffer (1 min), stained with haematoxylin (3–5 min), briefly immersed in acid alcohol for differentiation, transferred to a bluing solution to stabilize nuclear staining, and then rinsed thoroughly with water before subsequent processing. Counterstaining was performed in eosin (15 s) after 95% ethanol immersion (1 min). Dehydration was achieved through graded ethanol (100% I/II/III: 2 min each), cleared in xylene (I/II: 2 min each), and mounted with neutral gum. Stained sections were imaged under a light microscope for histological analysis.^47^

### 2.8 TUNEL Staining of Retinal Sections

Retinal cell death in vivo was evaluated using a TUNEL Cell Apoptosis Detection Kit (Servicebio, Cat# G1502). Briefly, enucleated eyeballs were fixed in 4% paraformaldehyde (PFA) at 4°C overnight, cryoprotected in a graded sucrose series (10%, 20%, and 30% sucrose in PBS), and embedded in OCT compound (Servicebio, Cat# G6059). Serial retinal cryosections (10 µm in thickness) were prepared using a cryostat (APV).

For TUNEL staining, frozen sections were baked at 37°C for 15 min, fixed in methanol for 1 h, and washed three times with PBS (pH 7.4). The sections were then permeabilized with Proteinase K working solution (diluted 1:40 in PBS) at 37°C for 10 min, followed by incubation in Equilibration Buffer at room temperature for 10 min. Subsequently, sections were incubated with the TUNEL reaction mixture containing recombinant TdT enzyme, fluorophore‐dUTP, and reaction buffer (mixed at a volume ratio of 2:5:50) in a humidified chamber at 37°C for 1 h in the dark. Cell nuclei were counterstained with DAPI staining solution (Servicebio, Cat# G1012) for 8 min at room temperature in the dark. Slides were mounted using an anti‐fluorescence quenching mounting medium (Servicebio, Cat# G1401).

Whole‐section digital images were acquired using a panoramic digital slide scanner (Servicebio) and visualized using Saiviewer analysis software (Servicebio). For quantitative evaluation, a blinded observer randomly selected three non‐overlapping regions within the combined ganglion cell layer (GCL) and inner plexiform layer (IPL) region from each retinal section. The GCL and IPL were analysed as a single combined region. The number of TUNEL‐positive cells was counted within a fixed area of 10 000 µm^2^ in each region and expressed as TUNEL‐positive cells per 10 000 µm^2^. The three regions selected from each retina were averaged to obtain one representative value for each animal. Three animals were analysed per experimental group (n = 3 animals/group), and the animal‐level values were used for statistical analysis.

### 2.9 Periodic acid‐Schiff (PAS)/haematoxylin staining

Mouse retinas were fixed in 4% neutral buffered formalin for 24 h at 4°C, then digested in 3% trypsin (Servicebio, G4004) at 37°C for 18–22 h until complete tissue dissociation was achieved. The digested retinas were gently agitated in PBS to separate the vascular networks from neuronal components, followed by meticulous microdissection under stereomicroscopic guidance. Isolated vascular networks were mounted on adhesive slides, air‐dried, and stained using the PAS/haematoxylin protocol (Servicebio, G1008). Briefly, slides were oxidized in 0.5% periodic acid (15 min), treated with Schiff's reagent (20 min), and counterstained with haematoxylin (5 min). Following sequential dehydration in graded alcohols and clearing with xylene, specimens were mounted with neutral resin.^48^ Vascular architecture was documented using a Nikon Eclipse Ni‐U microscope, with acellular capillaries quantified from 7 to 8 non‐overlapping fields per retina using ImageJ software.

### 2.10 In vivo fluorescence imaging and intraocular retention analysis

To evaluate the in vivo ocular distribution and retention kinetics of free curcumin and Cur‐NPs, DiR was used as a near‐infrared fluorescent tracer (excitation/emission = 748/780 nm). For the Cur‐NPs formulation, DiR was co‐encapsulated into the PLGA nanoparticles during preparation (DiR Cur‐NPs), whereas for the free curcumin group, DiR was physically mixed with unformulated curcumin immediately prior to administration (DiR Curcumin). Equivalent tracer amounts were incorporated into both formulations to ensure equal initial fluorescence intensity.

Mice were deeply anaesthetized and received a single intravitreal injection (1 µL per eye) of either DiR Curcumin or DiR Cur‐NPs. Longitudinal fluorescence imaging was performed at baseline (0 h, immediately post‐injection) and at 4, 8, 12, 24, 36, 48 and 72 h post‐administration using an IVIS Spectrum optical imaging system (PerkinElmer). All images were acquired under identical geometrical and exposure settings to ensure quantitative comparability across time points and treatment groups.

For signal quantification, regions of interest (ROIs) were standardized and manually delineated around the ocular region of each animal using Living Image software. The fluorescent signal within the ocular ROI was quantified as total radiant efficiency, expressed in units of (p/s/(µW/cm^2^). Background fluorescence from non‐injected anatomical regions was subtracted to calculate the net signal intensity. The longitudinal decline in DiR‐derived total radiant efficiency was plotted to define the intraocular clearance kinetics and relative ocular retention profiles of both formulations over the 72 h observation period.

### 2.11 RT­qPCR

After RNA isolation, complementary DNA was synthesized using a commercially available reverse‐transcription kit (Roche), and the quantitative amplification of target genes was carried out. Real‐time PCR profiling was executed on an ABI 7500 Real‐Time PCR System (Applied Biosystems) using the SYBR Green Master Mix (Roche).^49^, ^50^ Gene transcript levels were quantified by the comparative Ct (2^−ΔΔCt^) method. The sequences of primers employed for each gene are detailed in Tables S1 and S2.

### 2.12 Western blotting

Total proteins were extracted from retinas and cell lysates and quantified using a BCA Protein Assay Kit (Pierce). Samples were then resolved by SDS‐PAGE and subsequently transferred onto PVDF membranes (EMD Millipore).^51^ The membranes were blocked for 1 h at room temperature and then incubated with the primary antibodies overnight at 4°C. The detailed technical parameters, dilutions, and commercial vendors for all primary antibodies investigated—including anti‐β‐actin, anti‐NLRP3, anti‐ASC, anti‐CAS1, anti‐IL‐1β, anti‐IL‐18, anti‐PDGFR‐β, anti‐PDGFR‐B, anti‐Ang2, anti‐TGF‐β, anti‐CD206, anti‐Arg‐1, anti‐CD86, anti‐iNOS, anti‐TNF‐α, anti‐VEGF, anti‐VEGFR1, anti‐VEGFR2, anti‐MMP2 and anti‐MMP9—are explicitly catalogued in Table S3. Subsequently, we applied horseradish peroxidase (HRP)‐linked secondary antibodies to the membranes, allowing a 1‐h incubation phase at ambient temperature. Immunoreactive proteins were detected using enhanced chemiluminescence (ECL) reagents (EMD Millipore) and captured with a Tanon 5200 Multi Chemiluminescent Imaging System (Tanon).^52^, ^53^ Densitometric analysis was performed with ImageJ software (NIH), and β‐actin was used as the internal reference for normalization.

### 2.13 Cell culture and stimulation

Human retinal microvascular endothelial cells (HRMVECs, ZQY015) and human retinal microvascular pericytes (HRMVPCs, ZQY011) were purchased from Shanghai Zhong Qiao Xin Zhou Biotechnology. HRMVECs were maintained in endothelial cell medium (ECM; ScienCell), whereas HRMVPCs were cultured in Dulbecco's Modified Eagle Medium (DMEM; Gibco). Both culture media were supplemented with 10% fetal bovine serum (FBS) and 1% penicillin–streptomycin solution. Cells were maintained at 37°C in a humidified atmosphere containing 5% CO_2_. For subsequent assays, cells were seeded at the required density according to the specific experimental design.

After cell adhesion, PBS (vehicle control), free curcumin, Cur‐NPs (2.5 µg/mL), or other indicated treatments were applied according to the corresponding experimental protocol. Unless otherwise specified, the indicated treatments were initiated before hypoxia and maintained throughout the designated H/R experimental period. Cells were then subjected to hypoxia for 16 h followed by reoxygenation for 8 h (H/R). The hypoxic microenvironment was established using an AnaeroPack sealed chamber containing disposable oxygen‐absorbing catalyst sachets (AnaeroPack), with oxygen depletion monitored by indicator strips. For experiments involving NLRP3 activation, Nigericin was added during the final 1 h of the 8‐h reoxygenation period, as described in Section 2.15. RT‐qPCR and Western blot analyses were performed at the end of the 8‐h reoxygenation period.

### 2.14 Small Interfering RNA (siRNA) Transfection

To achieve genetic knockdown of NLRP3, human retinal microvascular endothelial cells (HRMVECs) were subjected to transient siRNA transfection. A splice‐specific small interfering RNA targeting human NLRP3 (designated as siNLRP3) and a non‐targeting scrambled control siRNA (siControl) were chemically synthesized by HaiXing Biosciences. The specific nucleotide sequences for siNLRP3 were as follows:

Sense: 5′‐GCUGCAGAUGGGAAUCUGGGAACCAGA‐3′;
Antisense: 5′‐UCUGGUUCCAGAUUCCAAUCUGCAGC‐3′.

HRMVECs were seeded into 6‐cm culture dishes and grown until they reached approximately 70%–80% confluency. For gene silencing, cells were transfected with siRNA using Lipofectamine 3000 (Invitrogen) following the manufacturer's guidelines. In brief, siRNA and Lipofectamine 3000 were individually diluted in Opti‐MEM reduced‐serum medium (Gibco), then carefully mixed and incubated at room temperature for 15 min to form lipid–siRNA complexes before adding to the cells. The mixture was then added dropwise to the culture wells to achieve a final siRNA working concentration of 50 nM. Following an incubation period of 6 h at 37°C in a 5% CO_2_ atmosphere, the transfection medium was thoroughly removed and replaced with fresh complete growth medium. To validate the knockdown efficiency, parallel cultures were collected at 24 h post‐transfection for RT‐qPCR analysis and at 48 h post‐transfection for Western blot analysis. Based on these validation experiments, siRNA‐transfected HRMVECs were subsequently used for the corresponding mechanistic and functional experiments according to the experimental design. Specifically, siNLRP3‐ or siControl‐transfected HRMVECs were subjected to PBS or Cur‐NPs treatment followed by H/R, with or without Nigericin stimulation, as specified for each experiment. Subsequent functional assays included scratch‐wound healing, Transwell migration and endothelial tube formation assays.

### 2.15 Pharmacological Treatments and Rescue Assays

To systematically modulate the NLRP3 pathway, HRMVECs were subjected to selective pharmacological inhibition or activation assays. For targeted pathway inhibition, cells were treated with MCC950 (Selleckchem), a selective small‐molecule NLRP3 inhibitor, at a final concentration of 5 µM for 24 h, either alone or in combination with Cur‐NPs (2.5 µg/mL), according to the corresponding experimental design. For pharmacological gain‐of‐function and rescue experiments, siControl‐ or siNLRP3‐transfected HRMVECs were subjected to the indicated PBS or Cur‐NPs treatment before H/R exposure. To pharmacologically activate the NLRP3 inflammasome, Nigericin (5 µM; GLBiol) was added during the final 1 h of the 8‐h reoxygenation period. Thus, Nigericin served as a secondary NLRP3‐activating challenge following H/R rather than as an independent stimulation condition. Experimental groups receiving Nigericin underwent the same 16‐h hypoxia followed by 8‐h reoxygenation protocol as their corresponding non‐Nigericin groups, with Nigericin introduced only during the final 1 h of reoxygenation. Following the respective pharmacological treatments, cells were collected at the designated experimental endpoints for downstream molecular analyses, including RT‐qPCR and Western blotting, or were used for subsequent functional assays, including scratch‐wound healing, Transwell migration and endothelial tube formation assays.

### 2.16 Cell Viability and Cytotoxicity Assay

Cell viability and baseline cytotoxicity were evaluated using a Cell Counting Kit‐8 (CCK‐8) assay (Dojindo Laboratories). The CCK‐8 experiments were performed under normoxic conditions and were designed to assess the baseline cytocompatibility of the tested formulations and mechanistic treatments, rather than to evaluate cell viability under H/R conditions. HRMVECs and HRMVPCs were seeded into 96‐well plates at 5 × 10^3^ cells/well and cultured for 24 h to allow cell adhesion.

For dose‐dependent cytotoxicity assessment, the culture medium was replaced with fresh medium containing PBS (vehicle control), free curcumin, or Cur‐NPs at final concentrations of 1.25, 2.5, 5, 10, 20, 40 or 80 µg/mL. Cells were maintained under normoxic conditions for 24 h following treatment. This dose range was used to evaluate the cytocompatibility of the formulations and to determine a suitable concentration for subsequent experiments.

To further assess the baseline cytocompatibility of the genetic and pharmacological perturbations used in the mechanistic experiments, HRMVECs were transiently transfected with siControl or siNLRP3 as described in Section 2.14. Following the indicated post‐transfection period, cells were treated with or without Cur‐NPs (2.5 µg/mL), and Nigericin (5 µM) was added for 1 h where indicated. These mechanistic cytotoxicity evaluations were performed under normoxic conditions without H/R exposure.

At the end of each designated treatment period, the culture supernatant was removed, and the cells were gently washed twice with PBS. A working solution containing 10% CCK‐8 reagent in fresh culture medium was then added to each well and incubated at 37°C for 1.5 h. The absorbance at 450 nm was measured using an Infinite 200 PRO microplate reader (Tecan). Cell viability was calculated according to the following equation:

$$Cellviability\left(\%\right)=\frac{OD_{\left(experiment\right)}-OD_{\left(blank\right)}}{OD_{\left(control\right)}-OD_{\left(blank\right)}}\;\times 100\%$$

where OD_experiment represents the absorbance of the corresponding treatment group, OD_control represents the absorbance of the PBS‐treated control group containing cells and culture medium, and OD_blank represents the absorbance of the medium‐only blank.

### 2.17 Cell migration assay

For evaluation of horizontal migration, a wound healing assay was performed under hypoxia/reoxygenation (H/R) conditions. HRMVECs and HRMVPCs in the exponential growth phase were seeded into 6‐well plates at 6 × 10^5^ cells/well and cultured for 24 h to reach full confluence. For experiments involving NLRP3 knockdown, only HRMVECs were transfected with siControl or siNLRP3 prior to the assay, as detailed in Section 2.14, whereas HRMVPCs were not subjected to siRNA transfection. Uniform linear scratches were generated using sterile pipette tips, followed by gentle washing with PBS to remove detached cells and cellular debris. Cells were then cultured in low‐serum medium containing PBS (vehicle control), free curcumin, or Cur‐NPs (2.5 µg/mL), according to the experimental design. The respective treatments were maintained throughout the subsequent H/R exposure. Ischemic injury was modelled by exposing cells to hypoxia for 16 h followed by reoxygenation for 8 h as described in Section 2.13. For designated NLRP3 activation/challenge groups, Nigericin (5 µM) was added during the final 1 h of the reoxygenation period. Wound closure was monitored by phase‐contrast microscopy at 0 h and at the end of the H/R exposure. For quantitative analysis, four randomly selected fields per well were examined, and wound closure was calculated as the percentage reduction in wound area relative to the initial area at 0 h.^54^

Vertical migration was evaluated using Transwell chambers (8‐µm pore size; Corning). For HRMVECs, the indicated siRNA transfections were performed prior to the assay as described in Section 2.14, whereas HRMVPCs were not subjected to siRNA transfection. Cells (4 × 10^4^ cells/well) suspended in 200 µL serum‐free medium were seeded into the upper chambers, while 500 µL complete medium was added to the lower chambers. Free curcumin or Cur‐NPs (2.5 µg/mL) was added to both the upper and lower compartments at the same final concentration, with PBS serving as the vehicle control. Maintaining the same concentration of each formulation in both compartments throughout the H/R exposure was intended to minimize concentration‐gradient‐dependent chemotactic effects. The Transwell assemblies were subjected to 16 h of hypoxia followed by 8 h of reoxygenation, with Nigericin (5 µM) added during the final 1 h of reoxygenation in the designated NLRP3 activation/challenge groups. Following completion of the H/R protocol, non‐migrated cells on the upper membrane surface were removed using cotton swabs. Migrated cells on the lower surface were fixed with 4% paraformaldehyde, stained with 0.1% crystal violet, and quantified by counting five randomly selected microscopic fields per insert for HRMVECs and four randomly selected microscopic fields per insert for HRMVPCs.^55^

### 2.18 Cell tube formation assay

Endothelial tube formation was assessed using a basement membrane matrix (BMM; BD Biosciences). BMM (50 µL/well) was added to 96‐well plates and polymerized at 37°C for 30 min. Prior to the assay, HRMVECs were transiently transfected with siRNAs (siControl or siNLRP3) as described in Section 2.14. Following siRNA transfection, cells were treated with PBS (vehicle control), free curcumin, or Cur‐NPs (2.5 µg/mL) and exposed to hypoxia for 16 h. Following hypoxic priming, HRMVECs (3 × 10^4^ cells/well) were harvested, resuspended in ECM containing the corresponding treatment, and seeded onto the polymerized BMM. Cells were incubated under normoxic reoxygenation conditions at 37°C with 5% CO_2_ for 8 h to allow capillary‐like network formation. The respective treatment conditions were maintained throughout the hypoxic exposure and subsequent reoxygenation/tube formation period. For designated NLRP3 activation/challenge groups, Nigericin (5 µM) was added during the final 1 h of the 8 h reoxygenation period. Tubular structures were visualized using an inverted microscope (Nikon Eclipse Ti). Four non‐overlapping microscopic fields were randomly selected and captured per well, and the number of junction points was quantified using ImageJ software as a quantitative measure of endothelial tube formation.^56^

### 2.19 Cellular uptake

Seeded into 6‐well plates at a density of 2.0 × 10^5^ cells/well, HRMVECs and HRMVPCs were maintained for 24 h until attaining 70%–80% confluence. The cell cultures were exposed to PBS (control), Cur‐NPs or curcumin solution for 2, 4 or 6 h at 37°C.^57^, ^58^ At each time point, cells were washed 3 times with PBS, fixed with 4% paraformaldehyde (10 min, RT), and washed again. Nuclear staining was performed using DRAQ5 (1:1000 in PBS, 800 µL/well, 15 min, dark). After staining, coverslips were mounted with antifade medium, and fluorescence images were captured with a Nikon Eclipse Ti inverted microscope. Three random fields per condition were captured for quantitative analysis.

### 2.20 Statistical analysis

Animals demonstrating poor health status were excluded from the study. To ensure objectivity, all experimental procedures were performed in at least triplicate, while investigators were kept blinded to the specific group assignments throughout both data acquisition and subsequent analysis. Statistical analyses were performed using SAS 9.0. Comparisons between two groups were evaluated using two‐tailed unpaired t‐tests, whereas multiple‐group comparisons were analysed using one‐way ANOVA followed by Tukey's post‐hoc test. For data involving two independent variables over time (such as in vivo fluorescence intensity kinetics), statistical comparisons were performed using two‐way ANOVA followed by Šídák's multiple comparisons test. A value of *p* < .05 was considered statistically significant. Data are presented as mean ± SEM.

## 3 RESULTS

### 3.1 Preparation and characterization of Cur‐NPs

**FIGURE 1:**
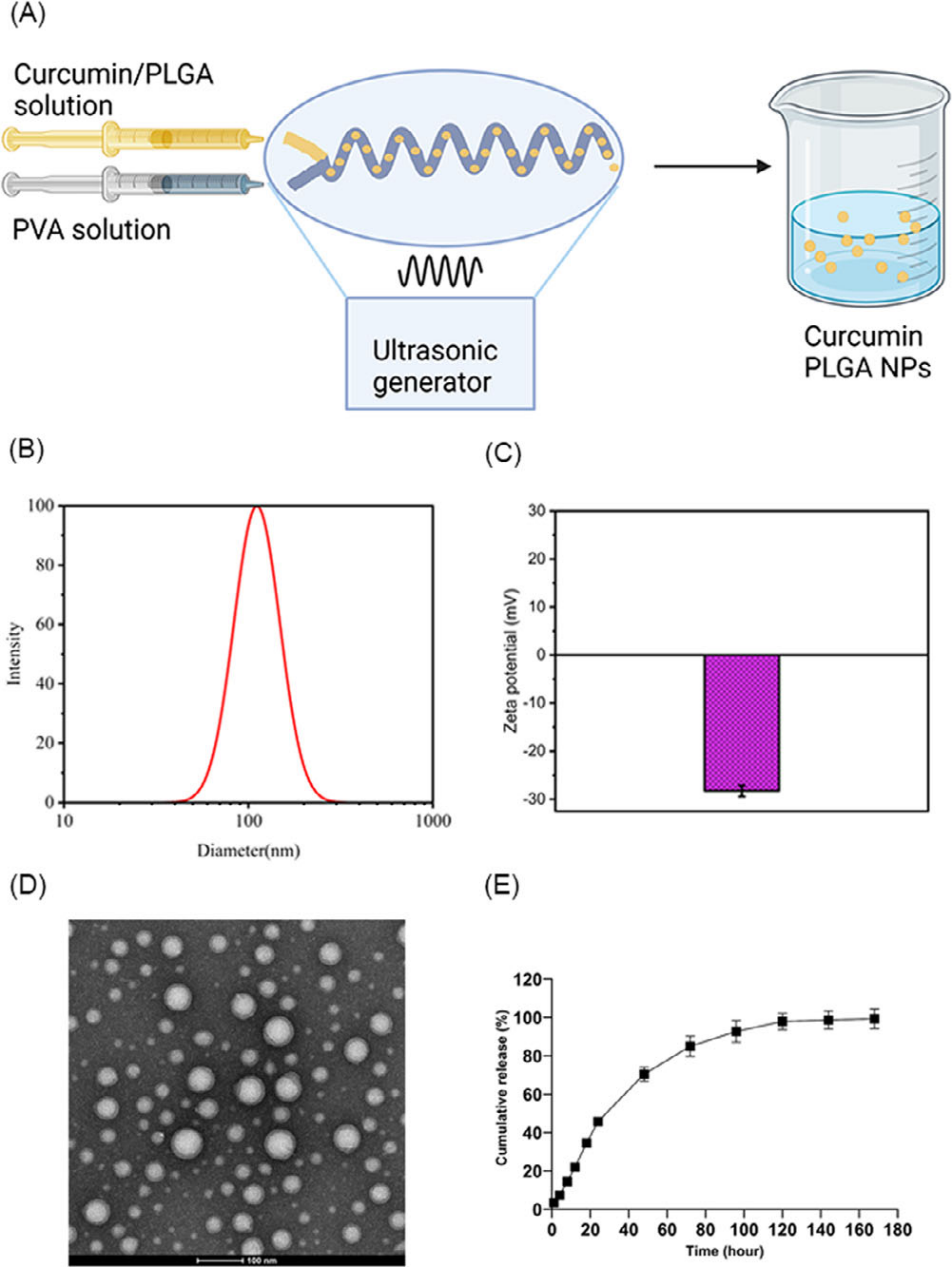
Preparation and characterization of Cur‐NPs. Schematic diagram of the ultrasonic microreactor device (A); size distribution (B) and zeta potential (C) of Cur‐NPs; transmission electron microscopy morphology of Cur‐NPs (D); cumulative release of curcumin from Cur‐NPs (E).

The Cur‐NPs were prepared via the emulsification method using an ultrasonic microreactor device. It is a controllable and scalable preparation process (Figure 1A).^59^, ^60^ Regarding the physical dimensions, Cur‐NPs exhibited a mean hydrodynamic diameter of 125.23 ± 1.78 nm alongside a polydispersity index (PDI) value of 0.143 ± 0.079 (Figure 1B). The zeta potential of the Cur‐NPs was around ‐28.3 mV, which suggested potential stability of the colloids (Figure 1C). The EE and DL of Cur‐NPs were 85.8% ± 1.3% and 8.2% ± 0.08%, respectively, indicating that the majority of the drug was successfully encapsulated. The TEM images showed that the Cur‐NPs had a round and smooth morphology with no aggregation between particles (Figure 1D). The in vitro release behaviour suggests sustained release of curcumin (Figure 1E).

### 3.2 Cur‐NPs Ameliorate Retinal Neovascularization and Vascular Pathology in vivo

**FIGURE 2:**
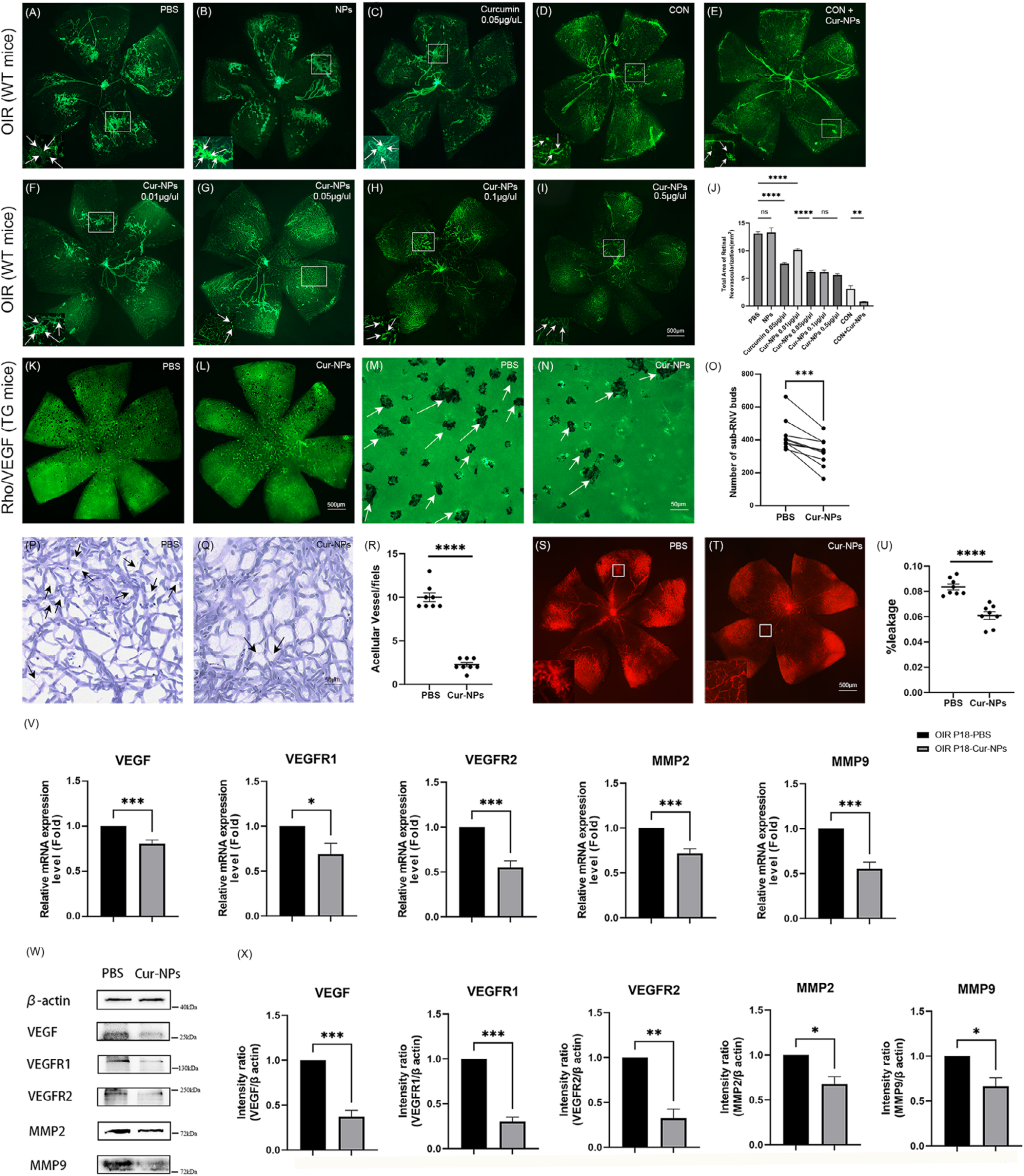
Cur‐NPs suppress pathological neovascularization and preserve retinal vascular integrity in vivo. (A–‐J) Representative retinal flat‐mount images of FITC‐IB4‐stained retinas from OIR (WT mice) at P18 following a single intravitreal injection at P12. The treatment groups were PBS (A), blank PLGA‐NPs (B), free curcumin (C), Conbercept (D), Conbercept + Cur‐NPs (E), Cur‐NPs at 0.01 µg/µL (F), Cur‐NPs at 0.05 µg/µL (G), Cur‐NPs at 0.1 µg/µL (H), and Cur‐NPs at 0.5 µg/µL (I). White arrows indicate pathological neovascular tufts. (J) Quantification of total retinal neovascularization area (*n* = 9–12 eyes per group). (K–O) Representative panoramic retinal flat‐mounts (K and L) and high‐magnification images (M and N) from rho/VEGF transgenic (TG mice) at P21 following intravitreal injection of PBS or Cur‐NP (0.05 µg/µL) at P12. White arrows in (M and N) indicate subretinal neovascular buds. (O) Quantification of subretinal neovascular lesions (*n *= 10 eyes per group). (P–R) Trypsin‐digested retinal vascular preparations from PBS‐ (P) and Cur‐NP‐treated (Q) OIR mice; black arrows indicate acellular capillaries. Quantification is shown in (R) (*n* = 8 random fields per retina). (S–U) Evans blue leakage assay in PBS‐ (S) and Cur‐NP‐treated (T) OIR retinas, with corresponding quantification of retinal vascular permeability (U) (*n* = 8 eyes per group). (V) Quantitative RT‐qPCR showing relative mRNA levels of *VEGF*, *VEGFR1*, *VEGFR2*, *MMP2* and *MMP9* in retinal tissues at P18 (*n* = 8 retinas per group). (W and X) Representative Western blot images (W) and densitometric quantification (X) of VEGF, VEGFR1, VEGFR2, MMP2 and MMP9 normalized to β‐actin (*n* = 3 independent biological replicates). Data are presented as mean ± SEM. *p < .05, **p < .01, ***p < .001, ****p < .0001. Statistical analysis was performed using one‐way ANOVA followed by Tukey's post‐hoc test for multi‐group comparisons (J) and two‐tailed Student's *t*‐tests for two‐group comparisons (O, R, U, V, X). Cur‐NPs, curcumin nanoparticles; CON, Conbercept; Curcumin, free curcumin solution; NPs, empty nanoparticles; OIR, oxygen‐induced retinopathy; P, postnatal day; RNV, retinal neovascularization; TG mice, Transgenic mice; WT mice, Wild‐Type mice.

To evaluate the therapeutic efficacy of Cur‐NPs, we employed two complementary animal models of ocular neovascularization: the oxygen‐induced retinopathy (OIR) model and the rho/VEGF transgenic mouse model (Figure 2).

#### 3.2.1 Suppression of retinal neovascularization

Retinal flat‐mount analysis demonstrated that Cur‐NPs and their combination with anti‐VEGF therapy effectively suppressed pathological retinal neovascularization (RNV) in OIR mice (Figure 2A–J). Empty PLGA‐NPs did not produce an appreciable therapeutic effect compared with the PBS control, whereas free curcumin (0.05 µg/µL) exhibited a modest inhibitory effect on RNV (Figure 2A–C). Cur‐NPs reduced the formation of neovascular tufts in a dose‐dependent manner over the concentration range of 0.01–0.5 µg/µL (Figure 2F–I). Notably, the 0.05 µg/µL dose produced a robust inhibitory effect, with no further apparent improvement at higher concentrations (Figure 2J). Monotherapy with the anti‐VEGF agent Conbercept (CON) also significantly reduced RNV; remarkably, combined administration of CON and Cur‐NPs (0.05 µg/µl) achieved the most pronounced suppression of neovascularization, resulting in a further significant reduction in pathological RNV area compared with CON monotherapy alone *(p* < .01; Figure 2D, E, and J). In rho/VEGF transgenic mice, Cur‐NP treatment (0.05 µg/µL) at P12 significantly reduced the number of subretinal neovascular buds by P21 compared with contralateral PBS‐injected eyes (Figure 2K–O).

#### 3.2.2 Improvement of vascular integrity and barrier function

Cur‐NPs (0.05 µg/µL) enhanced retinal vascular integrity, mitigated microvascular degeneration, and preserved histological architecture in OIR mice. The formation of acellular capillaries, a hallmark of ischaemic retinal damage, was prominent in PBS‐treated eyes but markedly reduced by Cur‐NP treatment (Figure 2P–R). Consistently, Evans blue leakage assays showed decreased pathological vascular permeability, indicating protection of the blood‐–retinal barrier (BRB) (Figure 2S–U).

#### 3.2.3 Downregulation of pro‐angiogenic signalling

To explore the underlying molecular mechanisms, we assessed key angiogenic factors in retinal tissues at P18. Evaluation of gene and protein expression via RT‐qPCR and Western blot showed that Cur‐NPs significantly reduced both the transcript and protein levels of VEGF, VEGFR1, VEGFR2, MMP2 and MMP9 (Figure 2V–X).

#### 3.2.4 Summary

Collectively, these multi‐level findings—encompassing structural vascular phenotypes, microvascular integrity, BRB barrier function, and downstream angiogenic signalling—demonstrate that Cur‐NPs effectively alleviate pathological ocular neovascularization and microvascular damage in vivo. Importantly, the significant additive reduction achieved by CON plus Cur‐NPs over CON monotherapy underscores the therapeutic potential of Cur‐NPs as a synergistic adjunctive strategy to complement standard anti‐VEGF regimens.

### 3.3 Cur‐NPs Preserve Retinal Architecture, Attenuate Apoptosis and Enhance Ocular Retention in vivo

**FIGURE 3:**
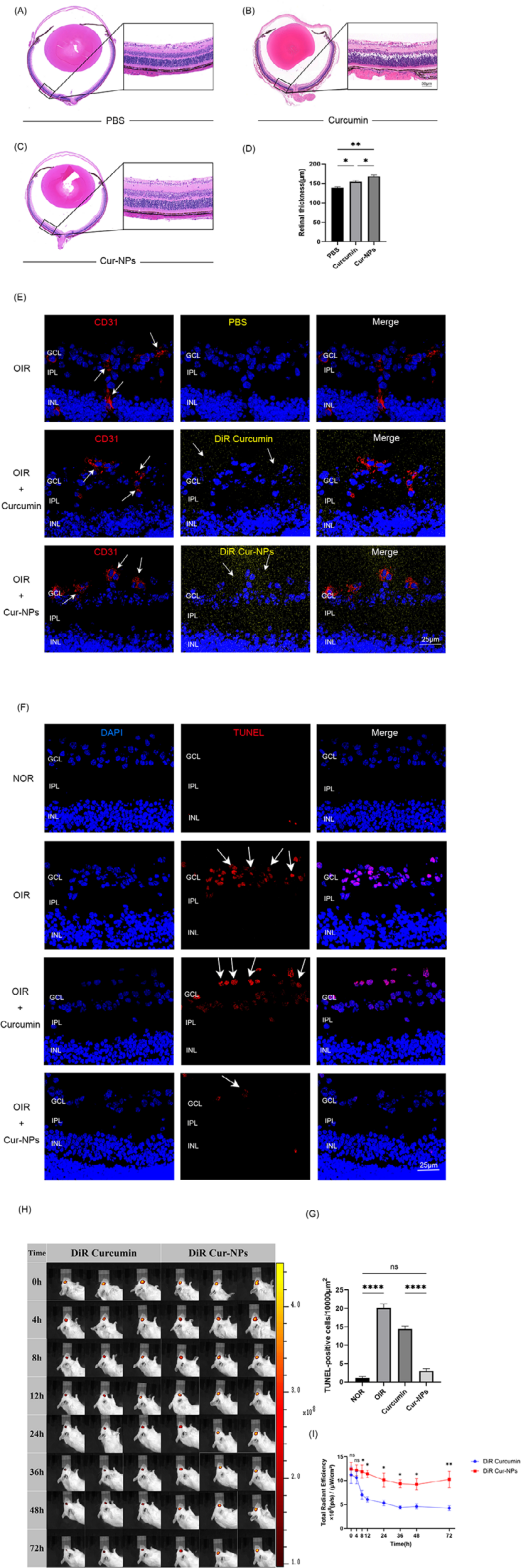
Retinal histological assessment, drug localization, apoptosis analysis and intraocular retention of free curcumin and Cur‐NPs in vivo. (A–D) Representative H&E‐stained retinal cross‐sections from OIR mice treated with PBS (A), free curcumin (B), or Cur‐NPs (C), and quantitative analysis of retinal thickness (D). Scale bar = 50 µm. (E) Representative fluorescence images of retinal cross‐sections following intravitreal administration of DiR‐loaded free curcumin or DiR‐loaded Cur‐NPs. CD31 immunofluorescence staining (red) was used to visualize retinal blood vessels, and DiR fluorescence (yellow) was used to track the distribution and localization of the respective formulations. Merged images show the spatial relationship between DiR fluorescence and CD31‐positive vascular structures. Scale bar = 25 µm. (F and G) Representative TUNEL‐stained retinal sections (F) and quantitative analysis of TUNEL‐positive cells (G) in NOR and OIR mice following intravitreal administration of free curcumin or Cur‐NPs. The ganglion cell layer (GCL) is indicated in the representative images. Scale bar = 25 µm. (H and I) Representative longitudinal in vivo IVIS images (H) and quantitative analysis of total radiant efficiency (I) following intravitreal administration of DiR‐loaded free curcumin or DiR‐loaded Cur‐NPs. Fluorescence signals were monitored at the indicated time points for up to 72 h after administration. Data are presented as mean ± SEM. *p < .05, **p < .01, ****p < .0001; ns, not significant. Statistical analysis for panels D and G was performed using one‐way ANOVA followed by Tukey's multiple‐comparisons test. Statistical analysis for panel I was performed using two‐way ANOVA followed by Šídák's multiple comparisons test. Cur‐NPs, curcumin nanoparticles; Curcumin, free curcumin solution; DiR Curcumin, DiR‐loaded free curcumin; DiR Cur‐NPs, DiR‐loaded curcumin nanoparticles; GCL, ganglion cell layer; INL, inner nuclear layer; IPL, inner plexiform layer; NOR, normal; OIR, oxygen‐induced retinopathy.

To systematically evaluate the potential advantages of nanoparticle encapsulation over free curcumin administration, we compared the retinal protective effects, DiR‐associated ocular distribution, and intraocular retention profiles of Cur‐NPs and free curcumin in the OIR model (Figure 3).

#### 3.3.1 Preservation of Retinal Histological Architecture

Histological examination by H&E staining revealed marked pathological alterations in the PBS‐treated OIR retinas, including disrupted retinal organization and reduced overall retinal thickness (Figure 3A). Treatment with free curcumin partially ameliorated these structural abnormalities (Figure 3B), whereas Cur‐NPs treatment resulted in more evident preservation of retinal lamination and tissue integrity (Figure 3C). Consistent with these morphological observations, quantitative analysis demonstrated that Cur‐NPs produced a more pronounced restoration of retinal thickness compared with the PBS‐treated group and showed greater protective effects than free curcumin (Figure 3D).

#### 3.3.2 Retinal Distribution of DiR‐Associated Fluorescence

To characterize the relative retinal distribution of the two formulations, DiR was used as a fluorescent tracer. For the Cur‐NPs formulation, DiR was encapsulated within the nanoparticles, whereas for the free curcumin formulation, an equivalent amount of DiR was mixed with free curcumin prior to intravitreal administration. Fluorescence microscopy of retinal cross‐sections revealed distinct distribution patterns between the two formulations. In the PBS‐treated control group, CD31‐positive pathological neovascular tufts were readily detectable, whereas no DiR‐associated fluorescence signal was observed. In contrast, the free curcumin group exhibited a relatively weak DiR‐associated fluorescence signal within the retinal tissue. Notably, the Cur‐NPs group exhibited a stronger and more widespread DiR‐associated fluorescence signal throughout the retina, with substantial spatial overlap with CD31‐positive vascular structures (Figure 3E).

#### 3.3.3 Attenuation of Retinal Cell Apoptosis

TUNEL staining was performed to further assess retinal cellular apoptosis following treatment. OIR mice exhibited prominent TUNEL‐positive signals, particularly within the ganglion cell layer (GCL), reflecting substantial retinal cellular injury under ischemic conditions (Figure 3F). Treatment with free curcumin partially attenuated the TUNEL‐positive signal. Notably, Cur‐NPs treatment resulted in a more pronounced reduction in TUNEL‐positive cells compared with free curcumin, indicating enhanced suppression of OIR‐associated retinal apoptosis by the nano‐formulated curcumin (Figure 3F,G).

#### 3.3.4 Prolonged Intraocular Retention of Cur‐NPs in vivo

To further investigate the intraocular retention characteristics of the two formulations, longitudinal IVIS imaging was performed for 72 h following intravitreal administration of formulations containing an equivalent amount of DiR (Figure 3H). The free curcumin formulation showed a relatively rapid decline in ocular DiR‐associated fluorescence, with a marked reduction in signal during the early observation period. In contrast, the Cur‐NPs formulation maintained a stronger fluorescence signal over time, with detectable DiR‐associated fluorescence persisting throughout the 72‐h observation period (Figure 3H). Quantitative analysis of total radiant efficiency further demonstrated a slower decline in fluorescence intensity in the Cur‐NPs group compared with the free curcumin group (Figure 3I), indicating prolonged ocular retention of the DiR‐associated signal in the Cur‐NPs formulation.

#### 3.3.5 Summary

Collectively, these findings demonstrate that Cur‐NPs provide greater retinal distribution of the DiR‐associated fluorescent tracer and prolonged ocular retention of the associated fluorescence signal compared with free curcumin, while more effectively preserving retinal tissue architecture and attenuating OIR‐associated retinal apoptosis. These findings support the potential advantages of Cur‐NPs as an intravitreal drug‐delivery formulation for sustained retinal protection.

### 3.4 Cur‐NPs Modulate the Retinal Microenvironment by Suppressing NLRP3 Activation, Macrophage Polarization and Pericyte Recruitment

To investigate how Cur‐NPs stabilize the retinal vasculature, we examined their impact on the retinal inflammatory and cellular microenvironment in OIR mice.

#### 3.4.1 NLRP3 inflammasome modulation

**FIGURE 4:**
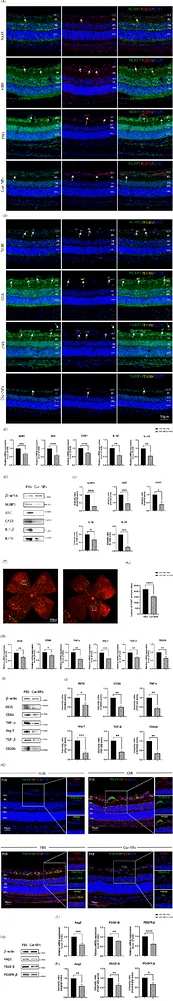
Cur‐NPs modulate the retinal microenvironment by suppressing NLRP3 activation, macrophage infiltration/polarization and pathological pericyte recruitment. (A and B) Representative confocal immunofluorescence images showing NLRP3 expression and cellular co‐localization in the retinal layers of NOR, OIR, OIR+PBS, and OIR+Cur‐NPs mice at P18. Retinal sections were double‐stained with the endothelial marker CD31 (red) or the macrophage marker F4/80 (yellow), with nuclei counterstained by DAPI (blue) (scale bar = 50 µm). (C–E) Quantitative analysis of NLRP3 inflammasome activation and expression in retinal tissue. (C) Relative mRNA expression levels of *NLRP3*, *ASC*, *CAS1*, *IL‐1β* and *IL‐18* in isolated retinal tissues determined by RT‐qPCR (*n* = 8 retinas per group). (D and E) Representative Western blot images (D) and densitometric quantification relative to β‐actin (*n* = 3 independent biological replicates per group) (E). (F and G) Evaluation of retinal macrophage infiltration. Representative panoramic flat‐mount images show F4/80 macrophage pooling (red) in OIR retinas at P18 following PBS or Cur‐NP treatment (F) (scale bar = 500 µm), with statistical quantification of total F4/80 cell counts per retina (G) (*n* = 12). (H–J) Phenotypic profiling of macrophage polarization. RT‐qPCR analysis of M1 markers (*iNOS*, *CD86*, *TNF‐α*) and M2 markers (*Arg‐1*, *CD206*, *TGF‐β*) (*n* = 8 retinas per group) (H), representative Western blots (I), and densitometric quantification (*n* = 3 independent biological replicates) (J). (K) High‐resolution confocal images of retinal cross‐sections at P18 illustrating pericyte coverage (PDGFR‐β, green) around neovascular structures (CD31, red), with nuclei counterstained by DAPI (blue) (scale bar = 50 µm). (L–N) Quantitative analysis of pericyte‐associated recruitment and microvascular stability regulators. RT‐qPCR of *Ang2*, *PDGF‐B*, and *PDGFR‐β* transcripts (*n* = 8 retinas per group) (L), representative Western blots (M), and densitometric protein quantification (*n* = 3 independent biological replicates) (N). Data are presented as mean ± SEM. *p < .05, **p < .01, ***p < .001, ****p < .0001. Statistical analysis was performed using a two‐tailed Student's *t*‐test for comparisons between the OIR+PBS and OIR+Cur‐NPs groups. Cur‐NPs, curcumin nanoparticles; GCL, ganglion cell layer; INL, inner nuclear layer; IPL, inner plexiform layer; OIR, oxygen‐induced retinopathy; ONL, outer nuclear layer; OPL, outer plexiform layer; P, postnatal day.

Immunofluorescence analysis showed that Cur‐NPs significantly attenuated the elevated NLRP3 expression and its co‐localization with CD31^+^ endothelial cells (Figure 4A) and F4/80^+^ macrophages in the retinal inner layers of OIR mice (Figure 4B). Consistently, RT‐qPCR and Western blot analyses confirmed the coordinated downregulation of the NLRP3 inflammasome components (NLRP3, ASC and CAS1) and their downstream pro‐inflammatory cytokines, IL‐1β and IL‐18, at both the mRNA and protein levels (Figure 4C–E).

#### 3.4.2 Macrophage infiltration and polarization

Whole‐mount retinal immunofluorescence revealed a significant reduction in F4/80^+^ macrophage infiltration following Cur‐NP treatment (Figure 4F,G). Molecular analyses indicated that both M1 indicators (iNOS, CD86, TNF‐α) and M2 markers (Arg‐1, CD206, TGF‐β) were robustly downregulated at the transcript and protein levels, demonstrating a comprehensive suppression of both the classical pro‐inflammatory (M1) and the pathological, pro‐angiogenic (M2) macrophage phenotypes (Figure 4H–J).

#### 3.4.3 Pericyte recruitment and vascular stabilization

Dual immunofluorescence staining for CD31 and PDGFR‐β revealed a pathological hyper‐recruitment and disorganized coverage of pericytes around the neovascular tufts in OIR and PBS‐treated eyes, which were distinctly normalized towards a restricted, physiological pattern upon Cur‐NP treatment (Figure 4K). This phenotypic stabilization was further corroborated by transcriptional and translational analyses, which confirmed the downregulation of Ang2, PDGF‐B and PDGFR‐β, indicating the restoration of vascular stability (Figure 4L–N).

#### 3.4.4 Summary

These results demonstrate that Cur‐NPs exert multi‐target protective effects by inhibiting NLRP3‐mediated inflammation, normalizing macrophage polarization, and regulating pericyte recruitment, thereby fostering a homeostatic retinal microenvironment that antagonizes neovascular progression.

### 3.5 Integrated Single‐Cell and Metabolomic Profiling Reveals Microglial Reprogramming and Metabolic Shifts Induced by Cur‐NPs

To thoroughly elucidate how Cur‐NPs modulate the retinal microenvironment, we performed integrated single‐cell transcriptomic (scRNA‐seq) and untargeted metabolomic analyses on OIR retinal tissues.

#### 3.5.1 Single‐cell transcriptomic atlas and microglial reprogramming

**FIGURE 5:**
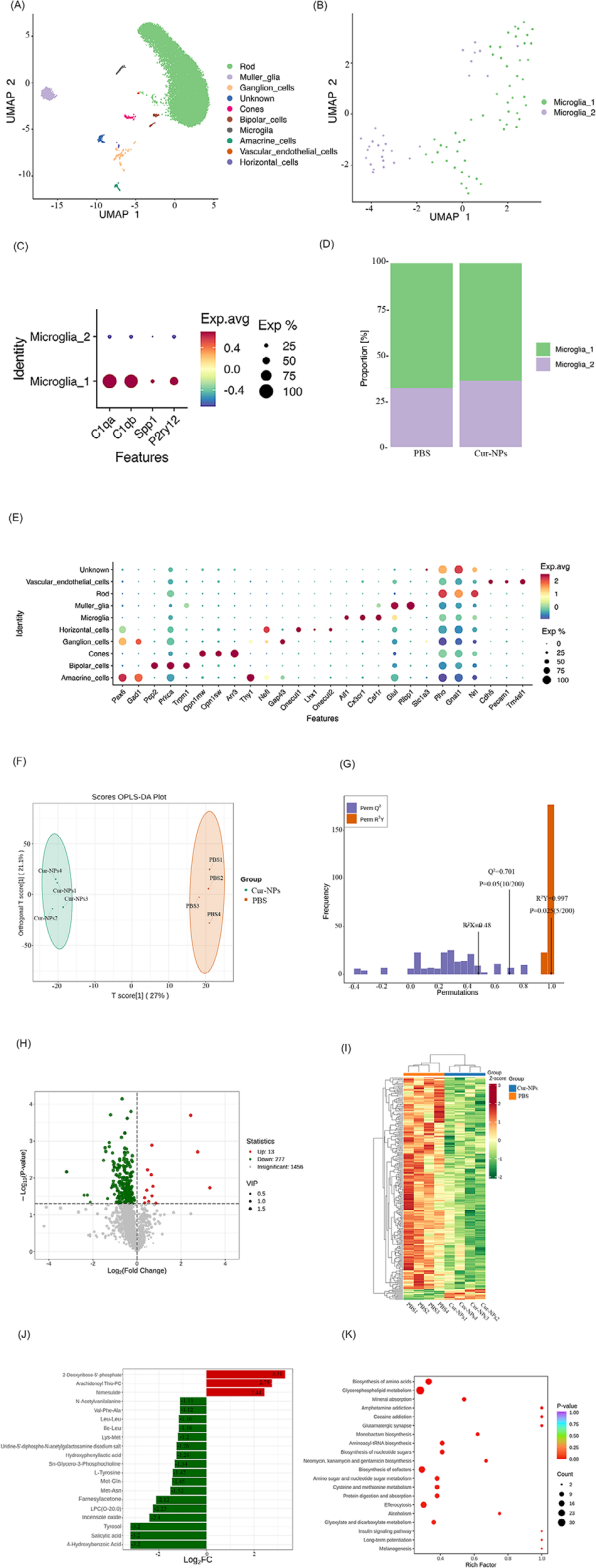
Integrated single‐cell transcriptomic and metabolomic profiling of Cur‐NP‐induced retinal microenvironmental reprogramming. (A) UMAP projection of 11 transcriptionally distinct retinal cell clusters, encompassing major neuronal, glial, and vascular lineages, color‐coded by cell type. (B) High‐resolution UMAP visualization of reclustered retinal microglial subpopulations, identifying two transcriptionally distinct subsets, Microglia_1 and Microglia_2. (C) Marker gene dot plot defining microglial subsets, showing enrichment of pro‐inflammatory genes (*C1qa*, *C1qb*) in Microglia_1 and homeostatic/regulatory markers (*Spp1*, *P2ry12*) in Microglia_2. (D) Bar plot showing proportional distribution shifts of Microglia_1 and Microglia_2 between PBS‐ and Cur‐NP‐treated groups. (E) Transcriptomic dot plot across all 11 retinal cell clusters validating canonical marker genes used for cluster identification. (F) OPLS‐DA score plot illustrating clear separation between the PBS and Cur‐NP cohorts (*n* = 4 independent biological replicates). (G) Cross‐validation permutation plot (200 iterations) showing R^2^Y and Q^2^ intercept values to assess model robustness and prevent overfitting. (H) Volcano plot of significantly altered retinal metabolites; red dots indicate upregulated metabolites, green dots indicate downregulated metabolites, and dot size corresponds to VIP values. (I) Hierarchical clustering heatmap showing group‐specific expression profiles of the top differentially expressed metabolites across biological replicates. (J) Top‐ranked differential metabolites are represented as horizontal bars, with red/green coloration indicating log_2_ fold change (log_2_FC) values. (K) KEGG pathway enrichment bubble plot for identified differential metabolites; dot size indicates metabolite counts per pathway, and colour represents enrichment *p*‐values. Cur‐NPs, curcumin nanoparticles; KEGG, Kyoto Encyclopedia of Genes and Genomes; OPLS‐DA, Orthogonal Projections to Latent Structures Discriminant Analysis; PBS, phosphate‐buffered saline; UMAP, Uniform Manifold Approximation and Projection; VIP, Variable Importance in Projection.

UMAP dimensionality reduction and SNN clustering of the scRNA‐seq data identified 11 distinct cell populations, which were rigorously annotated based on canonical retinal cell‐type marker genes (Figure 5A,E). Notably, subcluster analysis of the microglial population revealed two transcriptionally distinct activation states, designated as Microglia_1 and Microglia_2 (Figure 5B). Marker gene analysis showed that the Microglia_1 subset possessed a prominent pro‐inflammatory signature characterized by the heightened expression of C1qa, C1qb and Spp1 (Figure 5C). Crucially, quantitative composition analysis demonstrated that Cur‐NP treatment successfully remodelled this immune landscape, triggering a clear compositional shift characterized by a decreased proportion of the pro‐inflammatory Microglia_1 subset and a reciprocal expansion of the homeostatic Microglia_2 subset compared to PBS controls (Figure 5D).

#### 3.5.2 Untargeted metabolomic profiling and biomarker screening

To explore the biochemical milieu driving this microglial reprogramming, untargeted metabolomic analysis was conducted. Robust separation between the Cur‐NPs and PBS groups was demonstrated by OPLS‐DA score plots and validated via permutation testing (Figure 5F,G). Using stringent selection criteria (VIP > 1.5 and *p *< .05), we identified 290 significantly altered metabolites, visually resolved through volcano plot and hierarchical clustering heatmap analyses (Figure 5H,I). Among the top differentially expressed metabolites, Cur‐NP administration notably led to a profound downregulation of several specific components, including L‐tyrosine and sn‐glycero‐3‐phosphocholine (Figure 5J).

#### 3.5.3 KEGG pathway enrichment and metabolic‐inflammatory linkage

KEGG pathway enrichment analysis (Figure 5K) highlighted two pivotal metabolic pathways known to cross‐talk with the NLRP3 inflammasome cascade. First, within biosynthesis of amino acids, the depletion of L‐tyrosine represents a metabolic shift previously linked to the suppression of pro‐inflammatory macrophage/microglial activation. Second, alterations in glycerophospholipid metabolism suggest that Cur‐NPs modulate membrane lipid dynamics, which could mechanistically interfere with proper inflammasome assembly and subsequent pyroptotic cytokine release.

#### 3.5.4 Summary

Collectively, these multi‐omics findings demonstrate that Cur‐NP treatment induces a comprehensive reprogramming of the ischaemic retina, transitioning the microenvironment from a pro‐inflammatory microglial state into a favourable metabolic milieu that antagonizes activation of the NLRP3‐ASC‐CAS1 axis.

### 3.6 Cur‐NPs Exhibit Low Cytotoxicity in Retinal Vascular Cells and Enhanced Cellular Uptake in HRMVECs

To further translate our in vivo findings into cellular‐level evidence and to determine a suitable non‐cytotoxic working concentration for subsequent mechanistic studies, we evaluated the cytotoxicity and cellular uptake‐associated fluorescence of Cur‐NPs in retinal vascular cells.

#### 3.6.1 In vitro cytotoxicity and safe dosage screening

**FIGURE 6:**
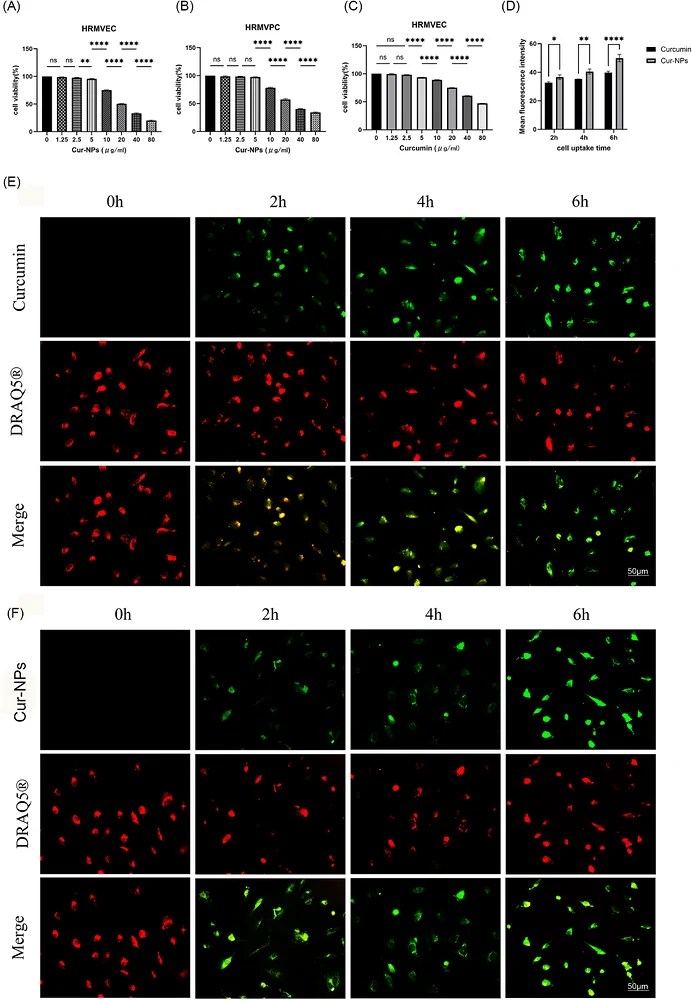
Cytotoxicity and cellular uptake of Cur‐NPs in retinal vascular cells. (A–C) Cell viability assessed by the CCK‐8 assay (*n* = 4 independent replicates per group). HRMVECs (A) and HRMVPCs (B) were incubated with Cur‐NPs at concentrations ranging from 0 to 80 µg/mL for 24 h. HRMVECs (C) were incubated with free curcumin at concentrations ranging from 0 to 80 µg/mL for 24 h as a reference treatment. (D) Quantitative analysis of intracellular fluorescence intensity comparing free curcumin and Cur‐NPs over time (*n* = 3 independent biological replicates). (E and F) Representative confocal fluorescence images showing intracellular uptake kinetics of free curcumin (E) and Cur‐NPs (F) in HRMVECs at 0, 2, 4, and 6 h. Top to bottom: green fluorescence of curcumin/Cur‐NPs, DRAQ5^®^ nuclear counterstain (red), and merged images. Data are presented as mean ± SEM. ns, not significant (*p* > .05), *p < .05, **p < .01, ****p < .0001. Statistical analysis was performed using one‐way ANOVA with Dunnett's post‐hoc test for dose‐response (A–C) and two‐way ANOVA with Sidak's multiple comparisons test for time‐course analysis (D). Cur‐NPs, curcumin nanoparticles; Curcumin, free curcumin solution; HRMVECs, human retinal microvascular endothelial cells; HRMVPCs, human retinal microvascular pericytes.

To determine a suitable concentration for subsequent in vitro mechanistic and functional studies, we first assessed the effects of free curcumin and Cur‐NPs on the viability of HRMVECs and HRMVPCs under normoxic conditions using the CCK‐8 assay (Figure 6A–C). In HRMVECs, neither free curcumin nor Cur‐NPs caused a significant reduction in cell viability at concentrations up to 2.5 µg/µL compared with the PBS‐treated control group. At 5 µg/µL, however, a mild reduction in HRMVEC viability was detected, while concentrations of 10 µg/µL and above produced a more pronounced, concentration‐dependent decrease in cell viability. In HRMVPCs, Cur‐NPs did not significantly affect cell viability at concentrations up to 5 µg/µL compared with the PBS control (*p* > .05). Based on these results, 2.5 µg/µL was selected as the working concentration of Cur‐NPs for subsequent in vitro mechanistic and functional experiments, as this concentration maintained favourable cell viability in both HRMVECs and HRMVPCs.

#### 3.6.2 Time‐Dependent Cellular Uptake and Intracellular Fluorescence Accumulation

To compare the cellular uptake‐associated fluorescence of free curcumin and Cur‐NPs, the intrinsic green fluorescence of curcumin was monitored in HRMVECs at 0, 2, 4 and 6 h after treatment. Cells were counterstained with the nuclear dye DRAQ5. Confocal fluorescence microscopy revealed minimal or undetectable green fluorescence at baseline (0 h), followed by a time‐dependent increase in intracellular green fluorescence in both groups (Figure 6E,F). Free curcumin‐treated cells exhibited a gradual increase in relatively diffuse cytoplasmic green fluorescence (Figure 6E). In contrast, Cur‐NPs‐treated cells showed stronger and more prominent intracellular green fluorescence, including punctate fluorescent structures, as early as 2 h, with progressively increased fluorescence intensity at 4 and 6 h (Figure 6F). Consistent with these microscopic observations, quantitative analysis of mean fluorescence intensity demonstrated that the Cur‐NPs group exhibited significantly higher cellular fluorescence than the free curcumin group at 2, 4 and 6 h (*p* < .05, *p* < .01, and *p* < .0001, respectively; Figure 6D). These findings indicate that Cur‐NPs facilitate greater cellular uptake‐associated fluorescence and intracellular accumulation of curcumin than free curcumin in HRMVECs.

#### 3.6.3 Summary

Collectively, these findings demonstrate that Cur‐NPs exhibited low cytotoxicity at the selected working concentration of 2.5 µg/µL in both HRMVECs and HRMVPCs, while producing stronger time‐dependent cellular uptake‐associated fluorescence and intracellular accumulation of curcumin in HRMVECs compared with free curcumin.

### 3.7 Cur‐NPs Suppress Angiogenic Functions and Downregulate Inflammatory and Angiogenic Mediators in Retinal Vascular Cells

**FIGURE 7:**
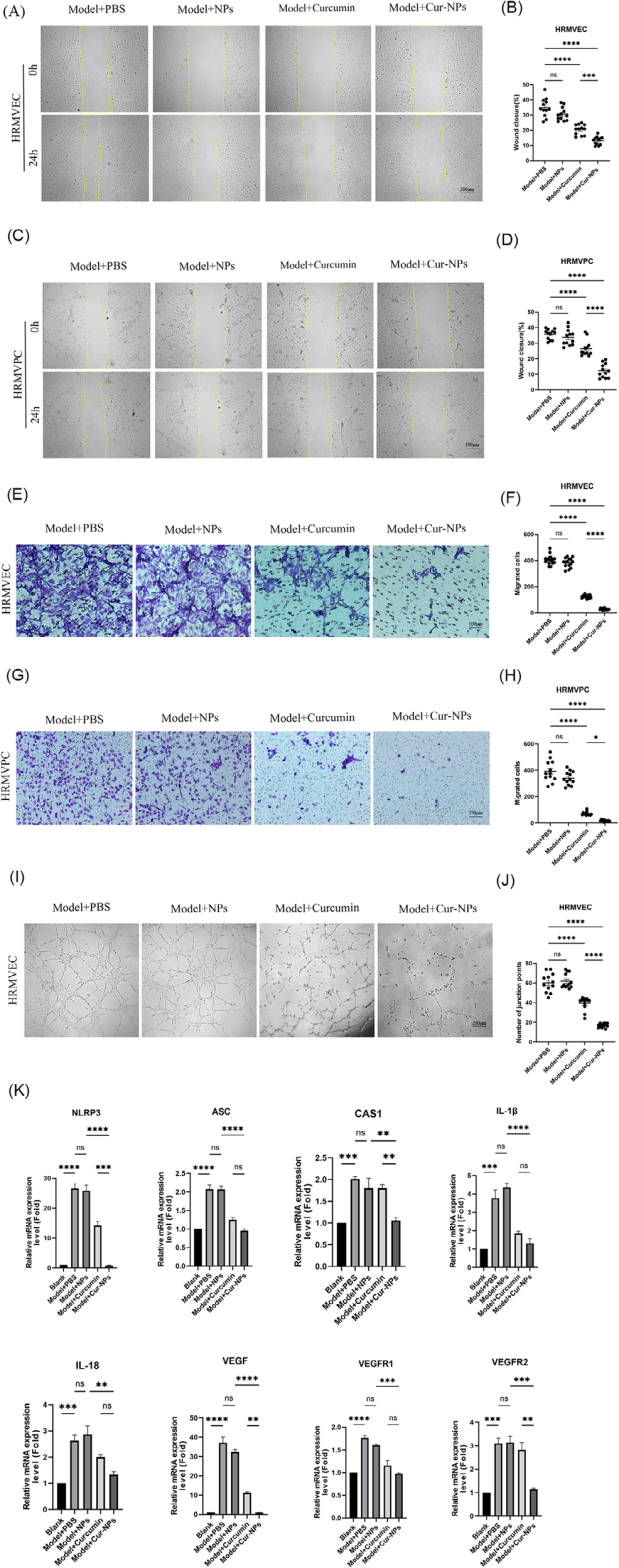
Cur‐NPs suppress angiogenic functions and downregulate inflammatory and angiogenic gene expression in retinal vascular cells. (A–D) Wound‐healing scratch assays assessing horizontal cell migration. Representative images and quantitative analysis of wound closure in HRMVECs (A and B; scale bar = 200 µm) and HRMVPCs (C and D; scale bar = 500 µm) following treatment with the indicated formulations under experimental H/R conditions. For each group, three independent wells were analysed, with four randomly selected non‐overlapping fields quantified per well (*n* = 3 independent wells per group). (E–H) Transwell migration assays assessing vertical cell migration. Representative images of crystal violet‐stained migrated cells and corresponding quantitative analysis in HRMVECs (E and F; scale bar = 100 µm) and HRMVPCs (G and H; scale bar = 250 µm) following treatment with the indicated formulations under experimental H/R conditions. For HRMVECs, three independent inserts were analysed, with five randomly selected fields quantified per insert; for HRMVPCs, three independent inserts were analysed, with four randomly selected fields quantified per insert (*n* = 3 independent inserts per group). (I and J) Matrigel tube formation assays assessing endothelial network formation in HRMVECs. Representative phase‐contrast images (I; scale bar = 200 µm) and quantitative analysis of total vascular junction points (J) following treatment with the indicated formulations under the experimental conditions. Three independent wells were analysed for each group, with four randomly selected non‐overlapping fields quantified per well (*n* = 3 independent wells per group). (K) RT‐qPCR analysis of inflammatory and angiogenesis‐associated gene expression in HRMVECs. Relative mRNA levels of *NLRP3*, *ASC, CAS1, IL‐1β, IL‐18, VEGF, VEGFR1* and *VEGFR2* were analysed in the Blank, Model+PBS, Model+NPs, Model+Curcumin, and Model+Cur‐NPs groups (*n* = 3 independent biological replicates per group). Data are presented as mean ± SEM. ns, not significant (*p* > .05), *p < .05, **p < .01, ***p < .001, ****p < .0001. Statistical analysis was performed using one‐way analysis of variance (ANOVA) followed by Tukey's post‐hoc multiple‐comparison test. Cur‐NPs, curcumin nanoparticles; HRMVECs, human retinal microvascular endothelial cells; HRMVPCs, human retinal microvascular pericytes; Model, hypoxia for 16 h followed by reoxygenation for 8 h (H/R).

To translate the protective effects observed in vivo into cellular‐level evidence, we investigated the effects of Cur‐NPs on key angiogenic behaviours and associated inflammatory and angiogenic mediators in retinal vascular cells. Functional assays were performed to assess endothelial and pericyte migration and endothelial tube formation, while RT‐qPCR was used to examine the expression of representative inflammatory and angiogenic genes in HRMVECs (Figure 7).

#### 3.7.1 Cur‐NPs Inhibit Endothelial and Pericyte Migration

We first evaluated the effects of free curcumin and Cur‐NPs on the migratory capacity of HRMVECs and HRMVPCs using wound‐healing and Transwell migration assays (Figure 7A–H). In the wound‐healing assay, the Model+PBS group exhibited substantial wound closure after H/R treatment, whereas empty NPs (Model+NPs) produced no significant difference compared with the Model+PBS group. Free curcumin reduced wound closure in both HRMVECs (Figure 7A,B) and HRMVPCs (Figure 7C,D), while Cur‐NPs produced a more pronounced reduction. Consistent with the wound‐healing results, Transwell assays showed that the number of migrated HRMVECs (Figure 7E, F) and HRMVPCs (Figure 7G, H) was markedly reduced following free curcumin treatment and was further decreased by Cur‐NPs. In contrast, no significant difference was observed between the Model+PBS and Model+NPs groups. These findings demonstrate that Cur‐NPs inhibit the migratory responses of both retinal endothelial cells and pericytes under H/R conditions.

#### 3.7.2 Cur‐NPs Inhibit Endothelial Tube Formation

We next examined the effects of Cur‐NPs on endothelial network formation using a Matrigel tube formation assay in HRMVECs (Figure 7I,J). Compared with the Model+PBS group, empty NPs did not significantly alter tube formation, whereas free curcumin reduced the formation of interconnected tubular structures. Cur‐NPs produced a further reduction in capillary‐like networks, with visibly fewer and less interconnected tubular structures than those observed in the free curcumin group (Figure 7I). Quantitative analysis of vascular junction points showed a significant reduction following Cur‐NP treatment compared with the Model+PBS, Model+NPs, and Model+Curcumin groups (Figure 7J). Together, these results indicate that Cur‐NPs suppress the formation of capillary‐like networks by HRMVECs under the experimental conditions.

#### 3.7.3 Cur‐NPs Downregulate Inflammatory and Angiogenic Gene Expression

To further characterize the molecular changes associated with these functional effects, we examined the mRNA expression of representative inflammatory and angiogenic mediators in HRMVECs by RT‐qPCR (Figure 7K). Compared with the Blank group, H/R treatment (Model+PBS) significantly increased the expression of several inflammatory and angiogenic genes, whereas empty NPs produced no significant changes compared with the Model+PBS group. Free curcumin partially attenuated these increases. Notably, Cur‐NPs further reduced the mRNA levels of *NLRP3* inflammasome‐associated genes, including *NLRP3, CAS1*, and *IL‐18*, compared with free curcumin, while also reducing the expression of *ASC* and *IL‐1β*. In parallel, Cur‐NPs decreased the expression of key angiogenesis‐associated genes, including *VEGF, VEGFR1* and *VEGFR2*, with the reductions in *VEGF* and *VEGFR2* being more pronounced than those observed following free curcumin treatment. These results indicate that Cur‐NPs suppress the transcriptional expression of key inflammatory and angiogenic mediators in HRMVECs, consistent with their inhibitory effects on vascular cell migration and endothelial tube formation.

#### 3.7.4 Summary

**FIGURE 8:**
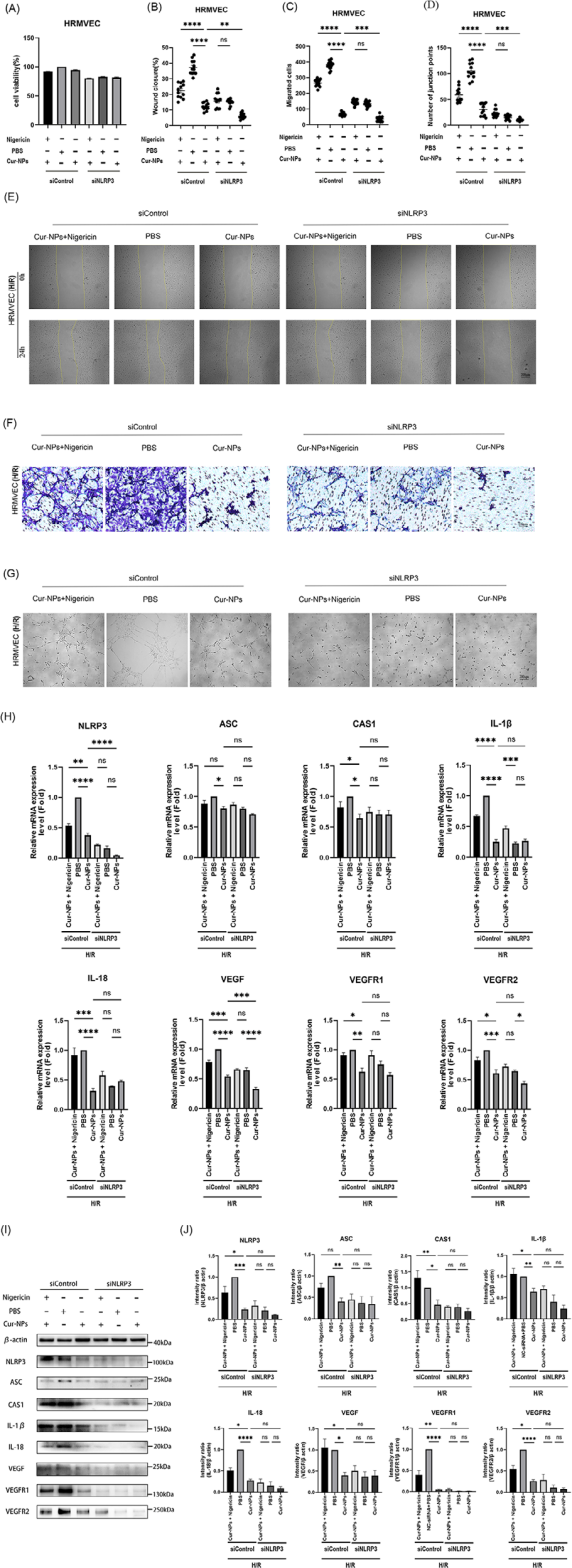
NLRP3 signaling contributes to the anti‐angiogenic effects of Cur‐NPs in HRMVECs. (A) Cell viability of HRMVECs after indicated genetic and pharmacological treatments was assessed using the CCK‐8 assay. (B–G) Functional evaluation of angiogenic responses under H/R conditions. Representative images and quantitative analyses are shown for wound‐healing scratch assays (B and E; scale bar = 200 µm), Transwell migration assays (C and F; scale bar = 100 µm), and Matrigel tube formation assays (D and G; scale bar = 200 µm). HRMVECs were subjected to H/R stimulation and treated with indicated combinations of Cur‐NPs, Nigericin‐mediated NLRP3 activation, or siRNA‐mediated NLRP3 knockdown (siNLRP3). For each group, three independent wells were analysed, with four randomly selected non‐overlapping fields quantified per well (*n* = 3 independent wells per group). (H) RT‐qPCR analysis of inflammatory and angiogenic gene expression under H/R conditions with indicated NLRP3 activation or knockdown treatments. Relative mRNA expression levels of inflammasome‐associated genes (*NLRP3, ASC* and *CAS1*), downstream inflammatory cytokines (*IL‐1β* and *IL‐18*), and angiogenic mediators (*VEGF*, *VEGFR1* and *VEGFR2*) were measured (*n* = 3 independent biological replicates per group). (I and J) Representative Western blot images (I) and corresponding densitometric quantification normalized to β‐actin (J; *n* = 3 independent biological replicates) of NLRP3‐associated inflammatory proteins and angiogenesis‐related signalling molecules under H/R conditions with indicated Cur‐NPs treatment, Nigericin‐mediated activation, or siRNA‐mediated NLRP3 knockdown. Data are presented as mean ± SEM. ns, not significant (*p* > .05), *p* < .05, *p < .01, **p < .001, ***p < .0001 (one‐way ANOVA with Tukey's post‐hoc test for multiple‐group comparisons). Cur‐NPs, curcumin nanoparticles; HRMVECs, human retinal microvascular endothelial cells; H/R, hypoxia for 16 h followed by reoxygenation for 8 h (H/R); siNLRP3, small interfering RNA targeting NLRP3; siControl, non‐targeting scrambled small interfering RNA.

Collectively, these findings demonstrate that Cur‐NPs inhibit key angiogenic cellular behaviours, including endothelial and pericyte migration and endothelial tube formation, and are associated with coordinated reductions in the expression of NLRP3 inflammasome‐associated and angiogenesis‐related mediators. The concurrent suppression of inflammatory and angiogenic gene expression provides a rationale for further investigating the potential involvement of NLRP3 signalling in the regulation of angiogenic responses and the therapeutic effects of Cur‐NPs, which was subsequently examined using genetic and pharmacological approaches (Figure 8).

### 3.8 Cur‐NPs Attenuate H/R‐Induced Angiogenic Responses Through Modulation of NLRP3‐Associated Signaling in HRMVECs

To further investigate whether NLRP3 signaling contributes to the anti‐angiogenic effects of Cur‐NPs, gain‐ and loss‐of‐function approaches were performed in HRMVECs under H/R conditions. NLRP3 activity was pharmacologically enhanced using Nigericin as a secondary inflammasome challenge during the final stage of reoxygenation, while NLRP3 expression was genetically suppressed using siNLRP3. The effects of these interventions on endothelial functional behaviours and inflammatory/angiogenic mediator expression were subsequently evaluated (Figure 8).

#### 3.8.1 Cell viability under genetic and pharmacological intervention conditions

To exclude potential cytotoxic effects caused by the combined treatments, cell viability was evaluated using CCK‐8 assays following siRNA transfection, Cur‐NPs treatment and Nigericin challenge. HRMVEC viability remained within an acceptable range across all experimental groups, indicating that the observed functional and molecular alterations were not primarily attributable to nonspecific cytotoxicity (Figure 8A).

#### 3.8.2 Cur‐NPs alleviate NLRP3 activation‐associated endothelial dysfunction under H/R conditions (Figure 8B–G)

To determine whether Cur‐NPs could maintain their protective effects under enhanced NLRP3 stimulation, HRMVECs were subjected to H/R treatment with or without Nigericin challenge during the final 1 h of reoxygenation. Under siControl conditions, Nigericin challenge further enhanced endothelial angiogenic behaviours, as reflected by increased wound closure (Figure 8B,E), elevated Transwell migration (Figure 8C,F), and increased endothelial network formation (Figure 8D,G) compared with the PBS‐treated H/R group. Treatment with Cur‐NPs markedly attenuated these Nigericin‐enhanced angiogenic responses, resulting in reduced endothelial migration and impaired tube formation.

To further assess the contribution of NLRP3 signalling, HRMVECs were transfected with siNLRP3 prior to H/R exposure. NLRP3 knockdown substantially reduced endothelial migration and tube formation under H/R conditions. Notably, additional Cur‐NPs treatment produced limited further inhibition in siNLRP3‐transfected cells, suggesting that NLRP3 signalling contributes to the cellular responses regulated by Cur‐NPs.

#### 3.8.3 Cur‐NPs suppress NLRP3‐associated angiogenic signalling under H/R conditions (Figure 8H–J)

We next examined whether modulation of NLRP3 signaling was associated with changes in inflammatory and angiogenic mediators. Under siControl conditions, Nigericin challenge during H/R significantly increased the expression of NLRP3‐associated inflammatory mediators, including NLRP3, ASC, CAS1, IL‐1β and IL‐18, as well as angiogenic factors including VEGF, VEGFR1 and VEGFR2 at both mRNA and protein levels (Figure 8H–J). Cur‐NPs treatment markedly reduced the expression of these inflammatory and angiogenic mediators despite Nigericin stimulation.

Consistent with the pharmacological intervention results, siNLRP3 significantly decreased the expression of inflammasome‐associated and angiogenesis‐related molecules under H/R conditions. Moreover, the additional inhibitory effect of Cur‐NPs was attenuated following NLRP3 knockdown, further supporting the involvement of NLRP3‐associated signalling in the anti‐angiogenic actions of Cur‐NPs.

#### 3.8.4 Summary

Collectively, these gain‐ and loss‐of‐function experiments demonstrate that Cur‐NPs effectively suppress H/R‐induced endothelial angiogenic responses under conditions of enhanced NLRP3 stimulation. Genetic inhibition of NLRP3 partially recapitulated the effects of Cur‐NPs and reduced the additional response to Cur‐NPs treatment, indicating that NLRP3‐associated signaling contributes to the regulation of Cur‐NPs‐mediated anti‐angiogenic activity. These findings further establish the involvement of NLRP3‐related inflammatory signaling in the protective effects of Cur‐NPs against pathological retinal vascular responses.

## 4 DISCUSSION

Our findings demonstrate that Cur‐NPs exert potent anti‐angiogenic effects in retinal neovascularization (RNV) primarily through targeted suppression of the NLRP3 inflammasome pathway. Pathological angiogenesis in ischaemic retinopathies, including diabetic retinopathy (DR) and retinopathy of prematurity (ROP), is currently managed by anti‐VEGF monotherapies; however, clinical non‐responsiveness, limited durability, and the risk of subretinal fibrosis highlight the need for upstream cascade‐targeted interventions. Importantly, persistent low‐grade inflammation has emerged as a critical driver of pathological vascular remodeling that may sustain angiogenesis despite VEGF blockade. By identifying the NLRP3–VEGF signalling axis, our study addresses this unmet need by demonstrating that NLRP3 functions as an upstream inflammatory regulator that maintains VEGF production and downstream VEGFR2 activation. Therefore, targeting the NLRP3–VEGF axis provides a complementary therapeutic strategy that simultaneously suppresses inflammatory amplification and angiogenic signalling, potentially overcoming limitations associated with VEGF‐only intervention.

At the tissue and cellular levels, Cur‐NPs effectively normalized the ischaemic retinal microenvironment. In the OIR model, Cur‐NPs preserved retinal structural integrity, reduced vascular leakage, and suppressed subretinal neovascular tufts. Pathological angiogenesis was associated with aberrant pericyte hyper‐recruitment and disorganized coverage around neovascular sprouts. Cur‐NPs restored a more physiological pattern of pericyte coverage, accompanied by transcriptional and translational downregulation of *Ang2*, *PDGF‐B* and *PDGFR‐β*. Given that chronic PDGF‐B/PDGFR‐β overactivation drives fibrovascular membrane formation and vascular destabilization, these findings indicate that Cur‐NPs promote vascular quiescence by suppressing pathological pericyte hyper‐recruitment rather than impairing physiological vessel stabilization.

Concurrently, Cur‐NPs suppressed reactive immune cell activation across the retinal layers. Rather than inducing a conventional macrophage/microglia phenotypic shift from M1 to M2, both classical pro‐inflammatory markers (iNOS^+^/CD86^+^) and tissue‐repair markers (Arg‐1^+^/CD206^+^) were synchronously downregulated, demonstrating a ‘dual‐suppression’ of reactive immune states. By dismantling the NLRP3 inflammasome assembly, Cur‐NPs reduce upstream danger signals that chronically drive microglial and macrophage activation, thereby restoring a homeostatic, quiescent immune environment and attenuating the downstream pro‐angiogenic cytokine cascade.

Integrated single‐cell transcriptomic and metabolomic analyses provided high‐resolution insight into this reprogramming. Microglial subpopulation analysis revealed a proportional reduction of the pro‐inflammatory Microglia_1 subset alongside preservation of the homeostatic Microglia_2, concordant with downregulation of canonical pro‐inflammatory markers (*C1qa*, *C1qb*) and enrichment of regulatory genes (*Spp1*, *P2ry12*). Parallel untargeted metabolomic profiling highlighted coordinated modulation of amino acid biosynthesis and glycerophospholipid metabolism. Given that intermediates of glycerophospholipid metabolism can function as endogenous danger signals that promote NLRP3 inflammasome assembly, the observed metabolic rewiring is consistent with the attenuation of upstream NLRP3 activation.^61^, ^62^, ^63^, ^64^ Collectively, these multi‐omics findings support a homeostatic mechanism whereby Cur‐NPs suppress pathological angiogenesis by disrupting the self‐sustaining feed‐forward loop in which metabolic stress reinforces NLRP3‐driven inflammation, thereby promoting VEGF‐mediated vascular remodelling.

Mechanistically, genetic and pharmacological perturbations confirmed the NLRP3‐ASC‐CASP1 axis as a primary functional target of Cur‐NPs, establishing a direct causal link to downstream angiogenic signalling. Cur‐NPs nearly abolished NLRP3 protein assembly, prevented Caspase‐1 cleavage, and reduced IL‐1β/IL‐18 maturation, which sequentially translated into diminished VEGFR2 activation and impaired capillary‐like network formation in vitro. Crucially, this upstream‐to‐downstream regulatory hierarchy was definitively substantiated using pharmacological gain‐of‐function ‘rescue’ assays with Nigericin, a potent NLRP3 activator. Even under hyper‐stimulatory conditions, co‐treatment with Cur‐NPs effectively neutralized the inflammatory surge, blunting NLRP3 hyperactivation and preventing the aberrant upregulation of VEGF and VEGFR2. By demonstrating that Cur‐NPs can counteract a pre‐activated NLRP3 state, these findings elevate the mechanistic interpretation from parallel correlation to direct causal dependency, confirming that the anti‐angiogenic efficacy of Cur‐NPs is fundamentally mediated through upstream NLRP3 inhibition and subsequent suppression of VEGF‐driven vascular remodeling. This causal hierarchy highlights the decisive advantage of targeting NLRP3 over canonical downstream angiogenic pathways: while standard anti‐VEGF agents merely scavenge extracellular ligands without addressing the underlying microenvironmental insult, upstream NLRP3 blockade halts both primary cytokine release (IL‐1β, IL‐18) and secondary pro‐angiogenic synthesis at their origin, effectively preventing inflammatory rebound and persistent vascular leakage. Furthermore, in siNLRP3 loss‐of‐function models, Cur‐NPs produced no additional suppression, demonstrating a robust ‘ceiling effect’ and confirming the absolute genetic necessity of NLRP3 in mediating their efficacy. Conversely, co‐administration of Cur‐NPs with MCC950, a selective small‐molecule NLRP3 inhibitor, elicited additive suppression of endothelial migration, invasion and angiogenic marker expression. While MCC950 partially inhibited inflammasome activation, residual NLRP3 activity remained, which was further suppressed by Cur‐NPs, resulting in amplified downregulation of VEGF, VEGFR2, MMP2, and MMP9 at both mRNA and protein levels. This pharmacological additivity, when contrasted with target saturation in siNLRP3 cells, underscores the fundamental difference between genetic ablation and functional protein inhibition. While siNLRP3 depletes the available NLRP3 transcript pool, MCC950 primarily blocks inflammasome activation without eliminating the target protein itself, thereby leaving residual signaling activity. Consequently, the ability of Cur‐NPs to further dampen this remaining cascade does not imply an independent secondary target. These findings suggest that Cur‐NPs achieve a greater degree of functional suppression of residual NLRP3‐associated signaling than MCC950 alone, potentially owing to the enhanced intracellular delivery and prolonged retention characteristics conferred by the nanoplatform.

The use of a PLGA‐encapsulated curcumin formulation enables practical translation by overcoming the physicochemical limitations of free curcumin, including poor solubility, rapid metabolic clearance, and low intraocular bioavailability. Cur‐NPs exhibited enhanced intracellular uptake, formed a stable aqueous suspension compatible with intravitreal injection, and provided sustained release within the vitreous humour, prolonging intraocular residence and potentially improving dosing convenience and local cytotoxicity. From a translational perspective, this prolonged intraocular residence time combined with upstream NLRP3 inhibition addresses two major clinical limitations of current anti‐VEGF monotherapy: the burden of frequent intravitreal injections and the chronic inflammatory rebound that often accompanies drug clearance.

Future studies should employ conditional, cell‐type‐specific NLRP3 knockout models to dissect microglial versus endothelial contributions in vivo. In addition, high‐resolution characterization of the molecular interactions between Cur‐NPs and the NLRP3 pyrin domain using SPR or cryo‐EM, alongside systematic dose‐response and long‐term safety studies, will further refine the translational therapeutic potential.

In conclusion, Cur‐NPs suppress pathological retinal angiogenesis through a cascade‐targeted mechanism involving upstream NLRP3 inflammasome inhibition, normalization of pathological pericyte recruitment, and reprogramming of the reactive immune and metabolic microenvironment. By directly linking upstream inflammatory sensing to downstream vascular remodeling, the NLRP3–VEGF axis provides a biologically robust and clinically advantageous framework for potentially addressing mechanisms associated with incomplete anti‐VEGF responsiveness, mitigating inflammatory‐driven recurrence, and reducing treatment burdens. By disrupting the NLRP3‐driven inflammatory–angiogenic cascade at its upstream source, Cur‐NPs provide a mechanistically distinct and potentially complementary therapeutic approach to conventional anti‐VEGF strategies and highlight the therapeutic potential of nanomedicine‐based interventions for neovascular retinal diseases.

## 5 CONCLUSION

This study establishes Cur‐NPs as a cascade‐targeted therapeutic strategy for retinal neovascularization (RNV) through precise suppression of the NLRP3 inflammasome. Cur‐NPs exert potent anti‐angiogenic effects via a sequential regulatory hierarchy: (1) inhibition of upstream NLRP3 activation and subsequent reduction of pro‐inflammatory cytokines (IL‐1β, IL‐18); (2) attenuation of downstream angiogenic signalling, including VEGF, VEGFR2, MMP2 and MMP9; and (3) remodelling of the retinal cellular microenvironment, encompassing normalization of pathological pericyte recruitment and coordinated suppression of reactive macrophage/microglia states.

High‐resolution, integrated single‐cell transcriptomic and untargeted metabolomic analyses reveal that Cur‐NPs reprogram the ischaemic retinal microenvironment through coordinated modulation of microglial subpopulations and glycerophospholipid metabolism. This effectively disrupts the feed‐forward loop linking metabolic stress to NLRP3 inflammasome‐driven vascular pathology. Mechanistic validation using siRNA‐mediated NLRP3 silencing, pharmacological inhibition, and gain‐of‐function rescue experiments confirms NLRP3 as the principal functional target underlying the anti‐angiogenic activity of Cur‐NPs. The nanoplatform's superior intracellular delivery and prolonged retention characteristics likely contribute to more complete suppression of residual NLRP3 signalling compared with conventional small‐molecule inhibitors.

Encapsulation of curcumin within a biodegradable PLGA carrier via an advanced ultrasound‐microreactor system ensures enhanced cellular uptake, stable intravitreal dispersion, and sustained release, overcoming the solubility and bioavailability limitations of free curcumin. Collectively, these findings highlight Cur‐NPs as a versatile nanomedicine platform that integrates upstream inflammasome inhibition with downstream angiogenic regulation, providing a translationally promising, mechanistically unified approach for the treatment of neovascular retinal disorders. Future studies should focus on optimizing dosing strategies and evaluating translational durability to maximize clinical outcomes.

## AUTHOR CONTRIBUTIONS

**Siyu Jiang**: Writing—original draft, validation, methodology, investigation, formal analysis, data curation, conceptualization. **Ziwei Cheng**: Writing—original draft, validation, methodology, investigation, formal analysis. **Yujuan Cai**: Methodology, formal analysis. **Yanji Zhu**: Validation, methodology. **Nyamjargal Nyambayar**: Validation, methodology. **Ruijie Lin**: Formal analysis. **Li Zhang**: Methodology. **Jian Chen**: Writing—review & editing, methodology, conceptualization. **Bing Xie**: Writing—review & editing, supervision, funding acquisition, conceptualization. **Xiuping Chen**: Writing—review & editing, supervision, methodology, conceptualization.

## CONFLICT OF INTEREST STATEMENT

The authors declare no conflicts of interest.

## ETHICS STATEMENT

All experimental procedures involving animals were conducted in strict accordance with the Guide for the Care and Use of Laboratory Animals and were approved by the Scientific Investigation Board of Shanghai Jiao Tong University School of Medicine, Shanghai, China.

## Supporting information



Supporting Information

Supporting Information

Supporting Information

Supporting Information

Supporting Information

Supporting Information

## Data Availability

The data that support the findings of this study are available on request from the corresponding author.
