# Supplementary material for: Cascade‐targeted intervention of the NLRP3 inflammasome and downstream angiogenesis by curcumin nanoparticles for ocular neovascularization therapy
Source: Clin Transl Med. 2026 Sep 21;16(9):e70846. doi: 10.1002/ctm2.70846 (PMC13594779; doi:10.1002/ctm2.70846)
Supplement: Supplementary file 1 [file CTM2-16-e70846-s002.docx]

Supplementary Table 1 Mouse primer sequences for qRT-PCR.

| Accession | Gene | GI number | Forward primer  (5'-3') | Reverse primer  (5'-3') | Product length |
| --- | --- | --- | --- | --- | --- |
| NM_145827.4 | NLRP3 | 1327850469 | GTTCTGAGCTCCAACCATTCT | CACTGTGGGTCCTTCATCTTT | 104 |
| NM_023258.4 | ASC | 165377184 | GCTGAGCAGCTGCAAACGA | ACTTCTGTGACCCTGGCAATGA | 136 |
| NM_009807.2 | Caspase-1 | 86198304 | TGCTGTCCCTGTATGCCTCT | TTGATGTCACGCACGATTTC | 100 |
| NM_008361.4 | IL-1β | 921274059 | GGTGTGTGACGTTCCCATTA | ATTGAGGTGGAGAGCTTTCAG | 106 |
| NM_001357221.1 | IL-18 | 1269208321 | TGAGGCATCCAGGACAAATC | GTACTCATCGTTGTGGGAACA | 96 |
| NM_009505.4 | VEGFA | 160358802 | CAGGCTGCTGTAACGATGAA | AATGCTTTCTCCGCTCAGAA | 207 |
| NM_007426.4 | Ang-2 | 443609488 | GGCTCTCACTGATGGACTTATT | CTGCCACATTCTCTCTCTCTTT | 124 |
| NM_010228.4 | VEGFR-1 | 1387845378 | CACCACTTGCACACACAAAG | AGCCATTCTCACAGCCAATAA | 104 |
| NM_010612.3 | VEGFR-2 | 1388591291 | AGCAGGATGGCAAAGACTAC | TACTTCCTCCTCCTCCATACAG | 114 |
| NM_013599.5 | MMP9 | 2020930347 | TGCACTGGGCTTAGATCATTC | TGCCGTCTATGTCGTCTTTATTC | 101 |
| NM_008610.3 | MMP2 | 985801704 | GCTCTGTCCTCCTCTGTAGTTA | GGTACAGTCAGCACCTTTCTT | 104 |
| NM_008809.2 | PDGFR-β | 226371751 | CAGTTGCCTTACGACTCCAC | GCTACTTCTGGCTGTCGATTT | 173 |
| NM_011057.5 | PDGF-B | 2292593697 | GAGTGTGGGCAGGGTTATTT | GAATCAGGCATCGAGACAGAC | 105 |
| NM_007482.3 | Arg-1 | 158966684 | CCTGAAGGAACTGAAAGGAAAG | TTGGCAGATATGCAGGGAGT | 71 |
| NM_008625.2 | CD206 | 224967061 | GTGGTCCTCCTGATTGTGATAG | CACTTGTTCCTGGACTCAGATTA | 139 |
| NM_011577.2 | TGF-β | 930697458 | ACGCCTGAGTGGCTGTCTTTTGAC | GGGCTGATCCCGTTGATTTCCACG | 148 |
| NM_013693.3 | TNF-α | 518831586 | GAACTGGCAGAAGAGGCACT | AGGGTCTGGGCCATAGAACT | 202 |
| NM_019388.3 | CD86 | 161484598 | AGCACGGACTTGAACAACCA | TGTAAATGGGCACGGCAGAT | 190 |
| NM_010927.4 | iNOS | 927028884 | CTCACCTACTTCCTGGACATTAC | GCCTCCAATCTCTGCCTATC | 101 |
