# Supplementary material for: Cascade‐targeted intervention of the NLRP3 inflammasome and downstream angiogenesis by curcumin nanoparticles for ocular neovascularization therapy
Source: Clin Transl Med. 2026 Sep 21;16(9):e70846. doi: 10.1002/ctm2.70846 (PMC13594779; doi:10.1002/ctm2.70846)
Supplement: Supplementary file 2 [file CTM2-16-e70846-s003.docx]

Supplementary Table 2 Human primer sequences for qRT­PCR.

| Accession | Gene | GI number | Forward primer  (5'-3') | Reverse primer  (5'-3') | Product length |
| --- | --- | --- | --- | --- | --- |
| NM_001243133.2 | NLRP3 | 1784638489 | GAAGAGGAGTGGATGGGTTTAC | TCTGCTTCTCACGTACTTTCTG | 102 |
| NM_013258.5 | ASC | 1519246347 | TCCTGACGGATGAGCAGTA | GGCTGGTGTGAAACTGAAGA | 86 |
| NM_001257118.3 | Caspase-1 | 1519242352 | TCTACCTCTTCCCAGGACATTA | GGACTTTCAGTACCCTTTCCTC | 110 |
| NM_000576.3 | IL-1β | 1653962476 | CAAAGGCGGCCAGGATATAA | CTAGGGATTGAGTCCACATTCAG | 102 |
| NM_001562.4 | IL-18 | 1519312029 | CCAAGGAAATCGGCCTCTATT | CATACCTCTAGGCTGGCTATCT | 112 |
| NM_003376.6 | VEGFA | 1677500543 | TGGTGTCTTCACTGGATGTATTT | AGTCTCTCATCTCCTCCTCTTC | 99 |
| NM_002019.4 | VEGFR-1 | 229892268 | AGGATGTGGAAGAGCATTAGC | GAGTCCCAACTGGAGAAATACC | 102 |
| NM_002253.4 | VEGFR-2 | 1936483952 | AGCAGGATGGCAAAGACTAC | TACTTCCTCCTCCTCCATACAG | 116 |
| NM_004530.6 | MMP2 | 1519242484 | AGAGAACCTCAGGGAGAGTAAG | CCTCGAACAGATGCCACAATA | 99 |
| NM_004994.3 | MMP9 | 1519311730 | GAACTTTGACAGCGACAAGAAG | CGGCACTGAGGAATGATCTAA | 115 |
| NM_002609.4 | PDGFR-β | 1519315263 | GCTCACCATCATCTCCCTTATC | CTCACAGACTCAATCACCTTCC | 90 |
| NM_033016.3 | PDGF-B | 939106609 | CTGTTGAGGTGGCTGTAGATG | GATGAAAGGAACCAGAGGAAGAG | 105 |
| NM_001386337.1 | Ang-2 | 1894803083 | ATCAGGACACACCACGAATG | CATCCTCACGTCGCTGAATAA | 125 |
