# Supplementary material for: Cascade‐targeted intervention of the NLRP3 inflammasome and downstream angiogenesis by curcumin nanoparticles for ocular neovascularization therapy
Source: Clin Transl Med. 2026 Sep 21;16(9):e70846. doi: 10.1002/ctm2.70846 (PMC13594779; doi:10.1002/ctm2.70846)
Supplement: Supplementary file 3 [file CTM2-16-e70846-s004.docx]

Supplementary Table 3 Detailed specifications, working dilutions, and commercial sources of the primary antibodies used for Western blot analysis.

| Target Antigen | Host Species | Dilution Ratio | Manufacturer |
| --- | --- | --- | --- |
| β-actin | rabbit | 1:5000 | Cell Signaling Technology |
| NLRP3 | rabbit | 1:1000 | Santa Cruz Biotechnology |
| ASC | rabbit | 1:1000 | Santa Cruz Biotechnology |
| CAS1 | rabbit | 1:1000 | Cell Signaling Technology |
| IL-1β | mouse | 1:1000 | Abcam |
| IL-18 | mouse | 1:1000 | Abcam |
| PDGFR-β | rabbit | 1:1000 | Santa Cruz Biotechnology |
| PDGFR-B | mouse | 1:400 | Santa Cruz Biotechnology |
| Ang2 | rabbit | 1:1000 | Santa Cruz Biotechnology |
| TGF-β | rabbit | 1:1000 | Santa Cruz Biotechnology |
| CD206 | rabbit | 1:1000 | Santa Cruz Biotechnology |
| Arg-1 | rabbit | 1:1000 | Santa Cruz Biotechnology |
| CD86 | rabbit | 1:2000 | Santa Cruz Biotechnology |
| iNOS | rabbit | 1:1000 | Santa Cruz Biotechnology |
| TNF-α | rabbit | 1:1000 | Santa Cruz Biotechnology |
| VEGF | rabbit | 1:1000 | Santa Cruz Biotechnology |
| VEGFR1 | rabbit | 1:1000 | Santa Cruz Biotechnology |
| VEGFR2 | rabbit | 1:1000 | Santa Cruz Biotechnology |
| MMP2 | rabbit | 1:1000 | Abways |
| MMP9 | rabbit | 1:1000 | ABclonal |
