# Supplementary material for: Cascade‐targeted intervention of the NLRP3 inflammasome and downstream angiogenesis by curcumin nanoparticles for ocular neovascularization therapy
Source: Clin Transl Med. 2026 Sep 21;16(9):e70846. doi: 10.1002/ctm2.70846 (PMC13594779; doi:10.1002/ctm2.70846)
Supplement: Supplementary file 4 [file CTM2-16-e70846-s005.docx]

#### **Supplementary Results**

**Supplementary 1. Cur-NPs potentiate the anti-angiogenic effects of pharmacological NLRP3 inhibition.**

To evaluate the translational potential of combining nanomedicine with targeted small-molecule therapeutics, we investigated the effects of Cur-NPs and the highly selective NLRP3 inhibitor MCC950 in HRMVECs. CCK-8 profiling confirmed that all individual and combined treatments maintained high cell viability (>95%), indicating that the observed functional changes were not due to cytotoxicity (Supplementary Fig. 1A).

In functional vascular assays, monotherapy with either low-dose Cur-NPs (2.5 μg/ml) or MCC950 (5 μM) significantly reduced endothelial motility and capillary network stability. Their combined administration further enhanced these effects, producing a cumulative suppression of angiogenic behaviors. Specifically, the Cur-NPs + MCC950 combination increased scratch-wound healing arrest (Supplementary Fig. 1B, E), exacerbated blockades in Transwell chemotactic migration (Supplementary Fig. 1C, F), and markedly decreased Matrigel tube formation branch points compared to either single treatment (Supplementary Fig. 1D, G).

These results indicate that Cur-NPs can cooperate with pharmacological NLRP3 inhibition to achieve enhanced anti-angiogenic activity in HRMVECs.

**Supplementary 2. Combined pharmacological and nano-formulated intervention enhances suppression of the NLRP3–VEGF signaling cascade.**

To investigate the molecular basis underlying the enhanced anti-angiogenic activity observed following combined treatment with Cur-NPs and MCC950, we quantified key components of the NLRP3–VEGF signaling cascade. RT-qPCR and Western blot analyses revealed that Cur-NPs significantly reduced NLRP3 expression at both the transcript and protein levels (Supplementary Figure 2A, L). In contrast, MCC950 alone produced only a modest reduction in NLRP3 protein abundance (Supplementary Figure 2K, L). Notably, combined treatment resulted in the lowest NLRP3 expression among all experimental groups.

Consistent with this enhanced suppression at the upstream inflammasome node, the combination treatment further decreased the expression of downstream inflammatory mediators, including IL-1β and IL-18, compared with either monotherapy alone (Supplementary Figure 2D, E, O, P).

Moreover, this inhibitory effect extended to multiple downstream angiogenic regulators. The combined treatment group exhibited significantly lower mRNA and protein levels of VEGF, VEGFR1, VEGFR2, MMP2, and MMP9 than either the Cur-NPs or MCC950 groups alone (Supplementary Figure 2F–J, Q–U). Collectively, these findings demonstrate that concurrent administration of Cur-NPs and MCC950 produces a greater suppression of the NLRP3–VEGF signaling cascade than either intervention alone.

#### Figure Legends:

**Supplementary Figure 1. Cur-NPs potentiate the anti-angiogenic effects of pharmacological NLRP3 inhibition in HRMVECs.**

(A) Cell viability assessed by CCK-8 assay following treatment with PBS, Cur-NPs (2.5 μg/ml), MCC950 (5 μM), or their combination for 24h(n=4 wells/group).

(E) Representative images and (B) quantitative analysis of scratch-wound healing migration in HRMVECs at 0h and 24h after wounding (n=12 fields/group).

(F) Representative images and (C) quantitative analysis of Transwell migration assays showing crystal violet-stained migrated cells (n=15 fields/group).

(G) Representative images and (D) quantitative analysis of Matrigel tube formation assays showing endothelial capillary-like network formation and the corresponding number of junction points (n=12-15 fields/group).

Data represent mean ± SEM from three independent experiments. Statistical significance was determined by one-way ANOVA followed by Tukey’s multiple-comparisons test. ****P* < 0.001; *****P* < 0.0001.

Abbreviations: Cur-NPs, curcumin nanoparticles; HRMVECs, human retinal microvascular endothelial cells.

**Supplementary Figure 2. Combined treatment with Cur-NPs and MCC950 enhances suppression of the NLRP3–VEGF signaling cascade in HRMVECs.**

(A–J) RT-qPCR analysis of the relative mRNA expression levels of inflammasome-related genes (*NLRP3*, *ASC*, *CAS1*, *IL-1β*, and *IL-18*) and angiogenesis-associated factors (*VEGF*, *VEGFR1*, *VEGFR2*, *MMP2*, and *MMP9*) in HRMVECs following the indicated treatments (n = 3).

(K) Representative Western blot images showing protein expression of NLRP3, ASC, CAS1, IL-1β, IL-18, VEGF, VEGFR1, VEGFR2, MMP2, and MMP9.

(L–U) Densitometric quantification of protein expression normalized to β-actin (n = 3 independent biological replicates).

Combined treatment with Cur-NPs and MCC950 resulted in greater suppression of NLRP3 expression and downstream inflammatory and angiogenic mediators compared with either treatment alone.

Data are presented as mean ± SEM. Statistical significance was determined by one-way ANOVA followed by Tukey’s multiple-comparisons test. ns, not significant; **P* < 0.05; ***P* < 0.01; ****P* < 0.001; *****P* < 0.0001.

Abbreviations: Cur-NPs, curcumin nanoparticles.
